# Supplementary material for: A High-Dimensional Quantification of Mouse Defensive Behaviors Reveals Enhanced Diversity and Stimulus Specificity
Source: Curr Biol. 2020 Dec 7;30(23):4619–4630.e5. doi: 10.1016/j.cub.2020.09.007 (PMC7728163; doi:10.1016/j.cub.2020.09.007)
Supplement: Document S2. Article plus Supplemental Information [file mmc6.pdf]

Current Biology

Article

A High-Dimensional Quantification of Mouse Defensive Behaviors Reveals Enhanced Diversity and Stimulus Specificity

Highlights

- Robust 3D reconstruction is developed to quantify posture and movements
- Responses to visual and auditory threats encompass multiple motor actions
- Responses are more stimulus specific than apparent from locomotion
- Each stimulus evokes more than one distinct behavioral response

Authors

Riccardo Storchi, Nina Milosavljevic, Annette E. Allen, Antonio G. Zippo, Aayushi Agnihotri, Timothy F. Cootes, Robert J. Lucas

Correspondence

riccardo.storchi@manchester.ac.uk

In Brief

Storchi et al. extend the study of instinctive defensive behaviors beyond the simple quantification of the locomotor state. By developing a high-dimensional quantification of mouse postures and movements, they show that defensive responses to visual and auditory threats are more diverse and stimulus specific than previously envisaged.

Storchi et al., 2020, Current Biology *30*, 4619–4630
December 7, 2020 © 2020 The Authors. Published by Elsevier Inc.
https://doi.org/10.1016/j.cub.2020.09.007

CellPress

Current Biology

CellPress
OPEN ACCESS

Article

A High-Dimensional Quantification of Mouse Defensive Behaviors Reveals Enhanced Diversity and Stimulus Specificity

Riccardo Storchi,[1,4,*] Nina Milosavljevic,[1] Annette E. Allen,[1] Antonio G. Zippo,[2] Aayushi Agnihotri,[1] Timothy F. Cootes,[3] and Robert J. Lucas[1]
[1]Division of Neuroscience and Experimental Psychology, School of Biological Science, Faculty of Biology, Medicine and Health, University of Manchester, Manchester, UK
[2]Institute of Neuroscience, Consiglio Nazionale delle Ricerche, Milan, Italy
[3]Division of Informatics, Imaging & Data Science, School of Health Science, Faculty of Biology, Medicine and Health, University of Manchester, Manchester, UK
[4]Lead Contact
*Correspondence: riccardo.storchi@manchester.ac.uk
https://doi.org/10.1016/j.cub.2020.09.007

SUMMARY

Instinctive defensive behaviors, consisting of stereotyped sequences of movements and postures, are an essential component of the mouse behavioral repertoire. Since defensive behaviors can be reliably triggered by threatening sensory stimuli, the selection of the most appropriate action depends on the stimulus property. However, since the mouse has a wide repertoire of motor actions, it is not clear which set of movements and postures represent the relevant action. So far, this has been empirically identified as a change in locomotion state. However, the extent to which locomotion alone captures the diversity of defensive behaviors and their sensory specificity is unknown. To tackle this problem, we developed a method to obtain a faithful 3D reconstruction of the mouse body that enabled to quantify a wide variety of motor actions. This higher dimensional description revealed that defensive behaviors are more stimulus specific than indicated by locomotion data. Thus, responses to distinct stimuli that were equivalent in terms of locomotion (e.g., freezing induced by looming and sound) could be discriminated along other dimensions. The enhanced stimulus specificity was explained by a surprising diversity. A clustering analysis revealed that distinct combinations of movements and postures, giving rise to at least 7 different behaviors, were required to account for stimulus specificity. Moreover, each stimulus evoked more than one behavior, revealing a robust one-to-many mapping between sensations and behaviors that was not apparent from locomotion data. Our results indicate that diversity and sensory specificity of mouse defensive behaviors unfold in a higher dimensional space, spanning multiple motor actions.

INTRODUCTION

Mice are innately able to respond to changes in their sensory landscape by producing sequences of actions aimed at maximizing their welfare and chances for survival. Such spontaneous behaviors as exploration [1, 2], hunting [3, 4], and escape and freeze [5–8], although heterogeneous, share the key property that they can be reproducibly elicited in the lab by controlled sensory stimulation. The ability of sensory stimuli to evoke a reproducible behavioral response in these paradigms makes them an important experimental tool to understand how inputs are encoded and interpreted in the brain and appropriate actions selected [5, 8–10].

Realizing the full power of this approach, however, relies upon a description of evoked behaviors that is sufficiently complete to encompass the full complexity of the motor responses and to capture the relevant variations across different stimuli or repeated presentations of the same stimulus. Instinctive defensive behaviors, such as escape or freeze, have been defined on the basis of a clear phenotype—a sudden change in locomotion state. Thus, in the last few years, it has been shown that speed, size, luminance, and contrast of a looming object have different and predictable effects on locomotion [5–8]. Nevertheless, mice do more than run, and a variety of other body movements as well as changes in body orientation and posture could, at least in principle, contribute to defensive behaviors. In line with this possibility, a wider set of defensive behaviors, including startle reactions and defensive postures in rearing positions, have been qualitatively described in rats [11, 12]. However, until now, a lack of tools to objectively measure types of movement other than locomotion has left that possibility unexplored.

We set out here to ask whether a richer quantification of mouse defensive behaviors was possible and, if so, whether this could provide additional information about the relationship between sensation and actions. To this end, we developed a method

This is an open access article under the CC BY license (http://creativecommons.org/licenses/by/4.0/).

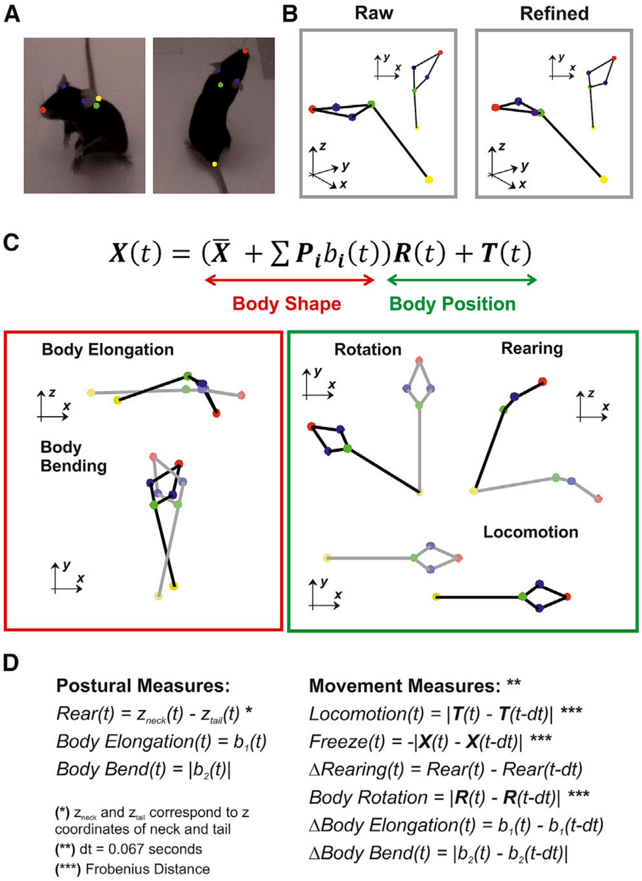

Figure 1. Reconstruction of Mouse Poses and Quantification of Postures and Movements

(A) Body landmarks are separately tracked across each camera. See Figure S1 for additional details on the 4-camera system.

(B) A raw 3D reconstruction is obtained by triangulation of body landmark positions (left panel). The raw reconstruction is corrected by applying our algorithm based on the statistical shape model as described in STAR Methods, Figure S2, and Videos S1 and S2. The refined 3D reconstruction (right panel) is then used for all the further analyses.

(C) The model expressed by Equation 1 allows for quantifying a wide range of postures and movements of which red and green boxes report some examples. The "body shape" components enable to measure changes in body shape, such as body elongation and body bending. For additional details, see STAR Methods section "Interpretation of the eigenposes" and Figure S3. The "body position" components enable to quantify translations and rotations in a 3D space.

(D) The full set of behavioral measures, divided into 3 postural measures and 6 movement measures, is expressed as function of the terms in Equation 1. For additional details, see STAR Methods section "Validation of the postural and movement measures" and Figure S4.

that enables to obtain a 3D reconstruction of mouse poses. We then used this method to generate a higher dimensional representation of mouse defensive behaviors that enabled to quantify a wide range of body movements and postures.

We found that defensive responses to simple visual and auditory stimuli encompass numerous motor actions and accounting for all those actions provides a richer description of behavior by increasing the dimensionality of behavioral representation. This increase provides an improved understanding of defensive behaviors in several respects. First, behavioral responses are more specific to distinct stimuli than is apparent simply by measuring locomotion. Second, higher specificity can be explained by the appearance of a richer repertoire of behaviors, with equivalent locomotor responses found to differ in other behavioral dimensions. Third, each class of sensory stimuli can evoke more than one type of behavior, revealing a robust "one-to-many" map between stimulus and response that is not apparent from locomotion measurements.

RESULTS

A Method for Quantifying Multiple Motor Actions

The first aim of this study was to develop a method that enables to obtain a 3D reconstruction of mouse poses. Five different landmarks on the mouse body (nose tip, left and right ears, neck base, and tail base; Figure 1A) were tracked using four cameras mounted at the top of an open-field arena that we used throughout the study (Figures S1A and S1B). The 3D pose of the animal was first reconstructed by triangulation of landmark coordinates across the four camera views (Figure 1B, raw; see STAR Methods section Reconstruction of 3D poses and Figures S1C–S1F for details). This initial reconstruction was then refined by using a method we established for this study (Figure 1B, refined; see STAR Methods section Reconstruction of 3D poses, Figure S2, and Video S1 for details). These pre-processing stages allowed us to describe, on a frame-by-frame basis, the mouse pose $\boldsymbol{X}$ as

$$\boldsymbol{X}(t) = \left(\overline{\boldsymbol{X}} + \sum_{i=1}^{N_{eigenposes}} \boldsymbol{P}_i b_i(t)\right)\boldsymbol{R}(t) + \boldsymbol{T}(t), \qquad \text{(Equation 1)}$$

where t represents the time of the current frame, $\boldsymbol{X}$ the coordinates of the body landmarks, $\overline{\boldsymbol{X}}$ the body coordinates of the mean pose, $\boldsymbol{P}_i$ the mouse eigenposes, b_i the shape parameters allowing to keep track of the changes in the body shape (Figure 1C, body shape), and $\boldsymbol{R}$ and $\boldsymbol{T}$ the rigid transformations (rotation and translation) encoding the animal's position in the behavioral arena (Figure 1C, body position). Both $\overline{\boldsymbol{X}}$ and $\boldsymbol{P}_i$ were obtained by training a statistical shape model (SSM) (Equation 3 in STAR Methods section Reconstruction of 3D poses) on a separate dataset of mouse poses. Those poses were first aligned, and a principal-component analysis (PCA) was performed to identify the eigenposes $\boldsymbol{P}_i$, i.e., the directions of largest variance with respect to $\overline{\boldsymbol{X}}$. Applying the SSM enabled to correct for outliers in the initial 3D reconstruction and to reduce high-dimensional noise while preserving meaningful changes in body shape (see STAR Methods section Validation of the 3D reconstruction and Figure S2 for details). The first two eigenposes captured, respectively, body elongation and bending (Figure 1C, body shape), two important descriptors of the mouse posture that explained, respectively, 43% and 31% of the variance associated with changes in body shape (see STAR Methods section Interpretation of the eigenposes, Figure S3, and Video S2 for details).

Based on this analytical description of the mouse pose, we developed two sets of measures to quantify distinct postures and movements. The first set of measures, rearing, body elongation, and body bending, allowed us to capture different aspects of the mouse posture (Figure 1D, postural measures). The

CellPress
OPEN ACCESS

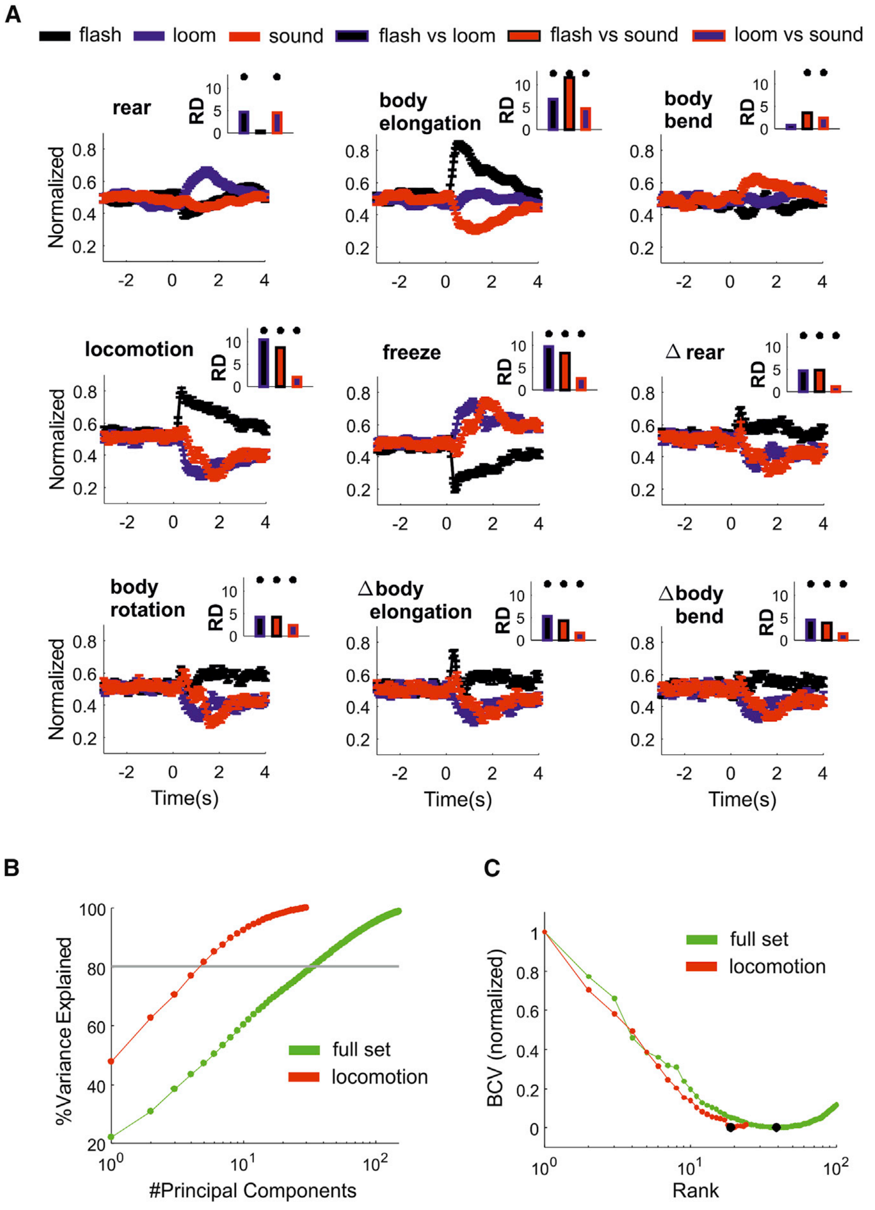

Figure 2. Multiple Motor Actions Are Involved in Sensory-Guided Behaviors

(A) Average response to the three classes of sensory stimuli (flash, loom, and sound) according to the postural and movement measures defined in Figure 1D. Error bars represent SEM (n = 172 for each stimulus class). Response divergence (RD) between pairs of stimuli is reported in insets (*p < 0.001 with shuffle test for RD).

(B) Percentage of variance explained as function of principal components for the full set of motor actions (green) and for locomotion only (red). The gray line indicates 80% explained variance.

(C) The minimum bi-cross validation error is used to quantify the rank of the full set and of locomotion only (respectively, rank = 19 and 39, marked by black dots).

second set, constituted by locomotion, freezing, rigid body rotation, and changes in rearing, body elongation, and body bending allowed us to capture different types of body movements (Figure 1D, movement measures). For all the analyses, the measures in Figure 1D were normalized and ranged in the interval [0, 1] (see STAR Methods section Normalization of the behavioral measures for details). These automatic measures were consistent with the human-based identification of walking, body turning, freezing, and rearing obtained from manual annotation of the behavioral videos (see STAR Methods section Validation of postural and movement measures and Figure S4 for details).

Measuring Multiple Motor Actions Provides a Higher Dimensional Representation of Behavior

We set out to investigate the extent to which our measures of postures and movements were involved in defensive behaviors. The animals were tested in an open-field arena, in which no shelter was provided. In order to capture a wide range of behavioral responses, we used three different classes of sensory stimuli: two visual and one auditory. Among visual stimuli, we selected a bright flash and a looming object. We have previously shown that these two stimuli evoke distinct and opposite behavioral responses, with the former inducing an increase in locomotor activity while the latter abolishes locomotion by inducing freezing behavior [7]. The auditory stimulus was also previously shown to induce defensive responses, such as freeze or startle [6, 13] (see STAR Methods sections Behavioral experiments, Visual and auditory stimuli, and Experimental set-up for details on sensory stimuli and experiments).

We separately averaged all trials according to stimulus class, and we found that all our measures were involved in defensive behaviors (Figure 2A). To estimate responses divergence (RD) across stimuli, we calculated the pairwise Euclidean distance between average responses, and we normalized this distance with that obtained by randomizing the association between stimuli and responses (Figure 2A, insets; see STAR Methods section Response Divergence for details). Across most measures (except rearing for loom and body bend for sound; see Figure 2A, insets), the average response to the flash clearly diverged from those elicited by other stimuli (RD = 6.28 ± 2.40 SD; p < 0.001 for n = 16 pairwise comparisons; shuffle test). Average responses to looming and sound were all significant but less divergent (Figure 2A, insets; RD = 2.52 ± 1.21 SD; p < 0.001 for n = 9 pairwise comparisons; shuffle test).

To determine whether the inclusion of all our measures of movements and postures, hereafter the "full set," increased the dimensionality of our behavioral description, we performed a PCA on the response matrix. For locomotion, each row of the

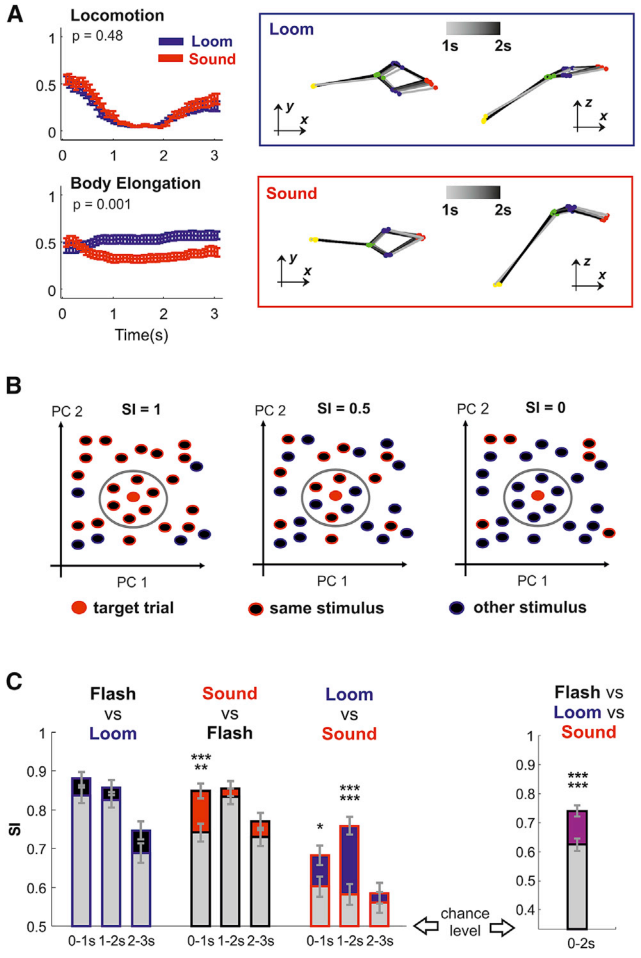

Figure 3. Higher Dimensionality Reveals Increased Stimulus Response Specificity

(A) Loom and sound could evoke an indistinguishable pattern of locomotion arrest shown in upper left panel (mean ± SEM; data from n = 37 and 31 trials for loom and sound). However, the pattern of body elongation was different across loom and sound (bottom left panel). A representative trial for loom (blue box) and for sound (red box) is reported in the right panels. Time progression is captured by the gray-to-black transition of the mouse body (poses sampled every 0.2 s between 1- and 2-s latency from stimulus onset). Note that different levels of body elongation can be observed from a side view in the z-x planes.

(B) On each trial, the specificity index (SI) was calculated as the number of neighbor responses to the same stimulus divided by the total number of neighbors. In this toy example, based on 2D responses (PC1 and PC2), we show a target trial for which the number of neighboring responses for the same stimulus changes across panels to obtain SI values of 1, 0.5, and 0.

(C) SI for pairs of stimuli (mean ± SD; n = 344 trials) measured with locomotion (gray bars) and for the full set (black, red, and blue). For additional details, see Figure S5.

(D) Same as (C) but for all stimuli (mean ± SD; n = 516 trials).

*p < 0.05; *****p < 0.0005; ******p < 0.0001.

response matrix represented a trial (n = 516 trials) and each trial contained 30 dimensions associated with the 0- to 2-s epoch of the locomotion time series (sample rate = 15 frames/s). For the full set, each trial contained 270 dimensions (30 time points × 9 measures). This analysis revealed that, for the full set, 34 principal components were required to explain >80% variance, although 5 dimensions were sufficient for locomotion alone (Figure 2B). In principle, the increase in dimensionality observed in the full set could be trivially explained by a disproportionate increase in measurement noise. To test for this possibility, we estimated the rank of the response matrix by applying the bi-cross validation technique [14] (see STAR Methods section Rank estimation for details). Consistent with the PCA analysis, we found that the rank of the full set was substantially larger, ∼2-fold (Figure 2C), indicating that the full set provided a genuine increase in dimensionality.

Higher Dimensionality Reveals Increased Stimulus Specificity in Defensive Behaviors

We then asked whether this increased dimensionality could capture additional aspects of stimulus-response specificity that could not be observed in locomotion. To account for the fact that evoked responses developed over time, we divided the responses into three consecutive epochs of 1-s duration according to their latency from the stimulus onset (“early”: 0–1 s; “intermediate”: 1–2 s; “late”: 2–3 s).

We first looked for a specific condition in which the same level of locomotion was expressed in response to two distinct sensory stimuli. A simple illustrative example, where locomotion largely fails to capture stimulus-response specificity, is the case in which both looming and sound induce a common freezing pattern that could be observed in a subset of trials (Figure 3A, top panels; see also Videos S3 and S4). In the intermediate response epoch, when freezing is strongest, locomotion “saturates” toward 0 in responses to both stimuli and thus provides no discrimination (p = 0.48; shuffle test for RD; n = 37 and 31 trials for loom and sound). However, stimulus specificity is apparent in the animal’s posture, as revealed by quantifying body elongation (Figure 3A, bottom panels p = 0.001; shuffle test for RD; n = 37 and 31).

To systematically compare stimulus-response specificity across all trials (n = 172 trials per stimulus) for the full set with the level of specificity revealed by locomotion alone, we developed a simple specificity index (SI). On an individual trial basis, SI identified, within a d-dimensional space, the k most similar behavioral responses across our dataset and quantified the fraction of those responses that were associated with the same stimulus. A toy example in which SI is calculated for $k = 6$ in a 2D dataset is depicted in Figure 3B. Thus, on a given trial, SI ranged from 0 to 1, in which 1 signifies all similar behavioral responses being elicited by the same stimulus, 0.5 similar responses being equally expressed for both stimuli, and 0 all similar responses being elicited by another stimulus (Figure 3B). For the real data, we used a weighted version of the SI index where the contribution of each neighbor response was inversely proportional to its distance from the target response (see STAR Methods section Stimulus-response specificity for a formal definition of the SI). The SI was applied to a principal component reduction of the response matrix (n = 15 and n = 15 × 9 = 135 time points for locomotion and the full set, respectively) and evaluated for pairwise comparisons between the 3 sensory stimuli. Because SI was dependent upon k and d, we systematically varied those parameters, and we recalculated SI for each parameter combination. Almost invariably, SI was maximized for $k = 1$ both for the full set and for locomotion only (Figure S5A). At least 5 principal components were typically required to maximize SI,

CellPress
OPEN ACCESS

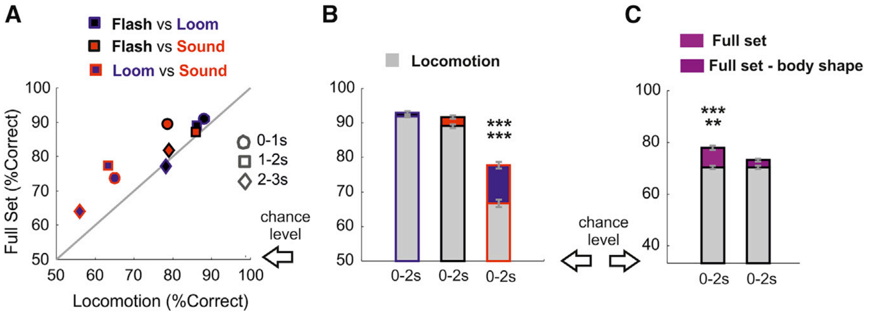

Figure 4. Higher Dimensionality Improves Stimulus Decoding

(A) Comparison between K-nearest neighbor (KNN) decoding performances (mean ± SD) based on the full set and on locomotion only. Pairwise comparisons are shown for flash versus loom (black-blue), sound versus flash (red-black), and loom versus sound (blue-red) across different response epochs (0 to 1 s, 1 to 2 s, and 2 to 3 s). For additional details, see Figure S6.

(B) Decoding performances (mean ± SD) of KNN decoding for 0- to 2-s response epochs.

(C) Same as (B), but decoding is performed across all stimuli for the full set (bright purple) and for a reduced set, in which we removed body elongation, body bending, Δbody elongation, and Δbody bending (dark purple). Locomotion is always displayed as gray bars.

*****p < 0.0005; ******p < 0.0001.

and the best value for *d* varied across different comparisons (Figure S5B). Therefore, low-dimensional responses (e.g., based on the first two components as in Figure S5C) failed to capture the full specificity of behavioral responses. Responses from the same animals were no more similar than those obtained from different animals because, for any given trial in the dataset, the most similar response rarely belonged to the same animal (n = 21; 10 trials out of 516 for full set and locomotion across all stimuli; p = 0.205, 0.957; shuffle test). Moreover, the distance between each target trial and its nearest neighbor was on average the same, irrespectively of whether they shared the same stimulus or not (Figure S5D).

We then compared SI between locomotion and the full set for $k = 1$ and the parameter *d* that returned the highest trial-averaged SI. We found no significant differences when comparing flash and loom (Figure 3C, blue bars; p = 0.0674, 0.2416, and 0.0701 for 0- to 1-s, 1- to 2-s, and 2- to 3-s epochs; sign test; n = 344 trials). However, the full set provided an increase in specificity for early responses when comparing flash and sound (Figure 3C, red bars; p = 0.0002, 0.4570, and 0.1980 for 0- to 1-s, 1- to 2-s, and 2- to 3-s epochs; sign test; n = 344 trials) and for the early and intermediate responses when comparing loom and sound (Figure 3C, red bars; p = 0.0254, 0, and 0.5935 for 0- to 1-s, 1- to 2-s, and 2- to 3-s epochs; sign test; n = 344 trials). For both the full set and locomotion, the highest SI values were observed either in the early or intermediate epoch of the response. We then set out to quantify the overall change in specificity. Compared with locomotion, the full set provided an overall ~40% increase in SI over chance levels (Figure 3D; p = 0; sign test; n = 516 trials).

To further test our conclusion that a higher dimensional description of behavior revealed increased stimulus-response specificity, we asked whether it improved our ability to predict the stimulus class based upon a mouse's behavior (i.e., whether higher dimensionality enables more accurate decoding of the stimulus). To this end, we applied a K-nearest neighbors (KNN) classifier because this algorithm utilizes that local information provided by the *k* neighbors and therefore represents a natural extension of the specificity analysis (see STAR Methods section Decoding analysis for details). Like the SI index, KNN decoding performances depended on the choice of *k* and *d*. Differently to what we observed for **SI**, where the index was maximized for $k = 1$, the best performances were obtained for larger values of *k*, indicating that multiple neighbors are required to reduce noise (Figure S6A). Similarly to SI analyses, high-dimensional responses substantially improved accuracy (Figure S6B).

Decoding performances were not significantly different for the full set and for locomotion when comparing flash versus loom (Figure 4A, black dots; p = 0.1130, 0.1384, and 0.6013 for 0- to 1-s, 1- to 2-s, and 2- to 3-s epochs; binomial test; n = 344 trials). However, the full set improved decoding of the early response for flash versus sound (Figure 4A, red dots; p = 0, 0.5356, and 0.26 for 0- to 1-s, 1- to 2-s, and 2- to 3-s epochs; binomial test; n = 344 trials) and across all epochs for loom versus sound (Figure 4A, blue dots; p = 0.0008, 0, and 0.0028 for 0- to 1-s, 1- to 2-s, and 2- to 3-s epochs; binomial test; n = 344 trials). These results were not specific for the KNN classifier because matching outcomes were obtained by using random forest (Figure S6C; flash versus loom: p = 0.4218, 0.2146, and 0.3671; flash versus sound: 0, 0.7528, and 0.5550; loom versus sound: 0, 0, and 0.0057; binomial tests; n = 344 trials). Focusing on the most informative 0- to 2-s epoch enabled to decode flash versus loom and flash versus sound with over 90% accuracy (respectively, 93% and 91.73%; Figure 4B, black and red bars), and the full set did not provide significant improvements over locomotion (p = 0.4901 and 0.1186; binomial test; n = 516 trials). However, when comparing loom versus sound, locomotion only allowed 66.78% accuracy although the full set provided 77.75% accuracy, a 65% improvement over chance level (p = 0.00001; binomial test; n = 516 trials). The full set also provided a 20.57% improvement over chance level when decoding was performed across the three stimuli (Figure 4C, purple bar; p = 0.0001; binomial test; n = 516 trials), which corresponded to an additional ~40 correctly decoded trials. Part of the increase in performance was granted by the information provided by changes in body shape (described in Figure 1D as body elongation, body bend, Δbody elongation, and Δbody bend) because removing those dimensions from the full set significantly degraded decoding performances (Figure 4C, dark purple bar; p = 0.0125; binomial test; n = 516 trials).

Higher Dimensionality Reveals a Larger Set of Defensive Behaviors

Our results indicate that the mapping between stimulus and behavioral response is more specific in a higher dimensional space. We next sought to describe the structure of this mapping. Specifically, we asked how many distinct behaviors are expressed in response to each stimulus. First, we clustered

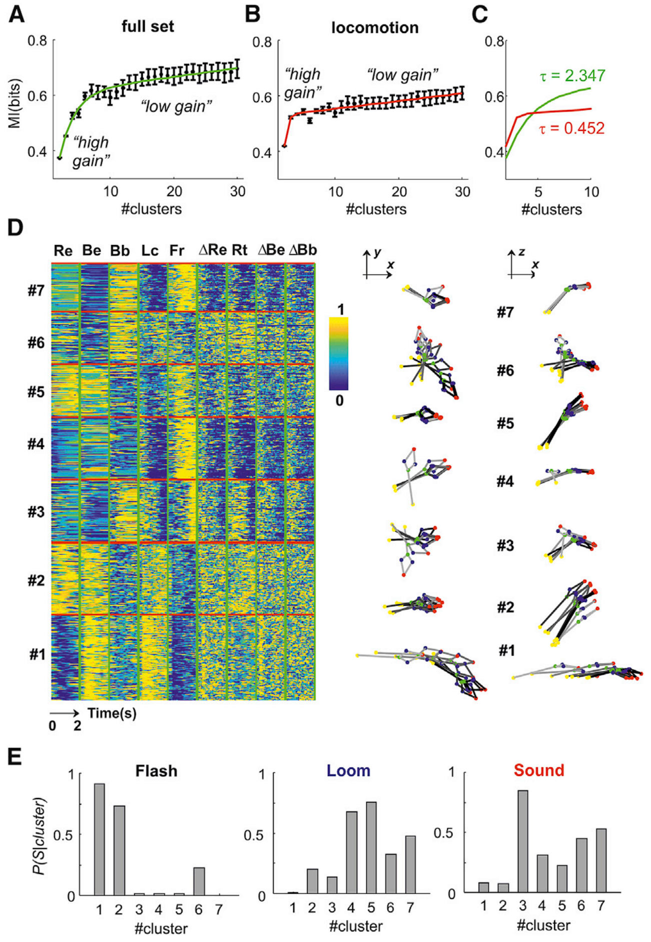

Figure 5. Higher Dimensionality Reveals a Larger Set of Sensory-Specific Behaviors

(A) Mutual information is estimated for the full set of motor actions as function of the number of clusters (mean ± SD; 50 repeats per cluster; at each repeat, the best of 100 runs was selected). Note an initial fast rise in MI ("high-gain" region in the plot) followed by a more gradual linear increase ("low-gain" region).

(B) Same as (A) but for locomotion only.

(C) Comparison between the exponential rise in MI for the full set of motor actions and for locomotion only. The exponential rise in MI, captured by the τ values, is slower for the full set, indicating that the high-gain domain encompasses a larger number of distinct clusters.

(D) Left panel shows the response matrix of the full dataset (n = 516 trials) partitioned into 7 clusters. The response matrix is obtained by concatenating all the postures and motor actions (ΔBb, Δbody bend; ΔBe, Δbody elongation; ΔRe, Δrear; Bb, body bend; Be, body elongation; Fr, freeze; Lc, locomotion; Re, rear; Rt, body rotation). Right panels show one representative trial for each cluster (10 poses sampled at 0.2-s intervals between 0- and 2-s latency from stimulus onset; time progression is captured by the gray-to-black transition).

(E) Conditional probability of stimulus class, given each of the clusters shown in (D). Flash, loom, and sound are reported, respectively, in left, middle, and right panel.

responses from all trials based upon similarity in motor actions. An important consideration in such a process is how many clusters to allow. We approached that problem by investigating the relationship between the number of clusters and the degree to which each cluster was restricted to a single stimulus (quantified as mutual information between stimulus and behavioral response). We focused on the interval 0–2 s because this epoch provided the best decoding results. Then, for each number of clusters, we estimated the mutual information (MI) between stimulus and behavioral response (see STAR Methods section Clustering and Information Analysis for details). By observing the increase in MI as function of the number of clusters, two distinct regions could be clearly delineated (Figure 5A, black error bars). For a small number of clusters, approximately between 2 and 7, we observed a "high gain" region where MI increases substantially for each additional cluster. Beyond this domain, the "high-gain" region was replaced by a "low-gain" region, where further increments in the number of clusters provided limited increments in MI. This analysis suggests 7 clusters as a reasonable trade-off between the need for a generalization of the behavioral responses and the granularity required to capture a large fraction of stimulus-specific information.

Our previous analyses suggested that the range of behaviors is larger when considering the full set versus locomotion alone (see, e.g., Figure 3A). To confirm that this was true, we applied the same clustering method to the locomotion data alone. A similar repartition into high- and low-gain regions was observed (Figure 5B, black error bars). However, the high-gain region domain appeared to be reduced to approximately 2 to 3 clusters, suggesting a reduction in the number of sensory-specific behavioral clusters. To more rigorously test whether this was the case, we fitted the relation between MI and the number of clusters k using the function

$$MI(k) = a\left(1 - e^{\frac{-k}{\tau}}\right) + bk, \qquad \text{(Equation 2)}$$

which incorporates a steep exponential component and a more gradual linear component (Figures 5A and 5B, fitting lines; see STAR Methods section Clustering and Information Analysis for details). These terms account, respectively, for the high- and the low-domain regions. We then used the exponential rise constant τ as a measure of the size of the high-domain region. We found that τ was indeed smaller for locomotion alone (Figure 5C), indicating that the full set of measures of postures and movements captures a larger number of sensory-specific behaviors.

Among the 7 behaviors revealed by our clustering of the full set, several motifs occurred (Figure 5D): fast sustained locomotion (cluster no. 1) or rearing (cluster no. 2) both accompanied by body elongation; body bending followed by delayed freeze (cluster no. 3); sustained freeze (cluster no. 4); transient freeze in rearing position (cluster no. 5); body bending and other rotations of the body axis, including frequent changes in rearing position (cluster no. 6); and sustained freeze in body bent positions (cluster no. 7). The flash stimulus evoked behaviors that were very specific for this stimulus (clusters no. 1 and no. 2; Figure 5E, left panel). The loom and sound stimuli evoked approximately the same set of behaviors, but, between the two stimulus classes, those behaviors were expressed in different proportions (Figure 5E, middle and left panel).

Distinct Behaviors Differ Both in Rate and Latency of Behavioral Primitives

Each of those 7 behaviors was composed of several basic motor actions and postures that we define as primitives. In principle, distinct behaviors could contain diverse sets of primitives and/

CellPress
OPEN ACCESS

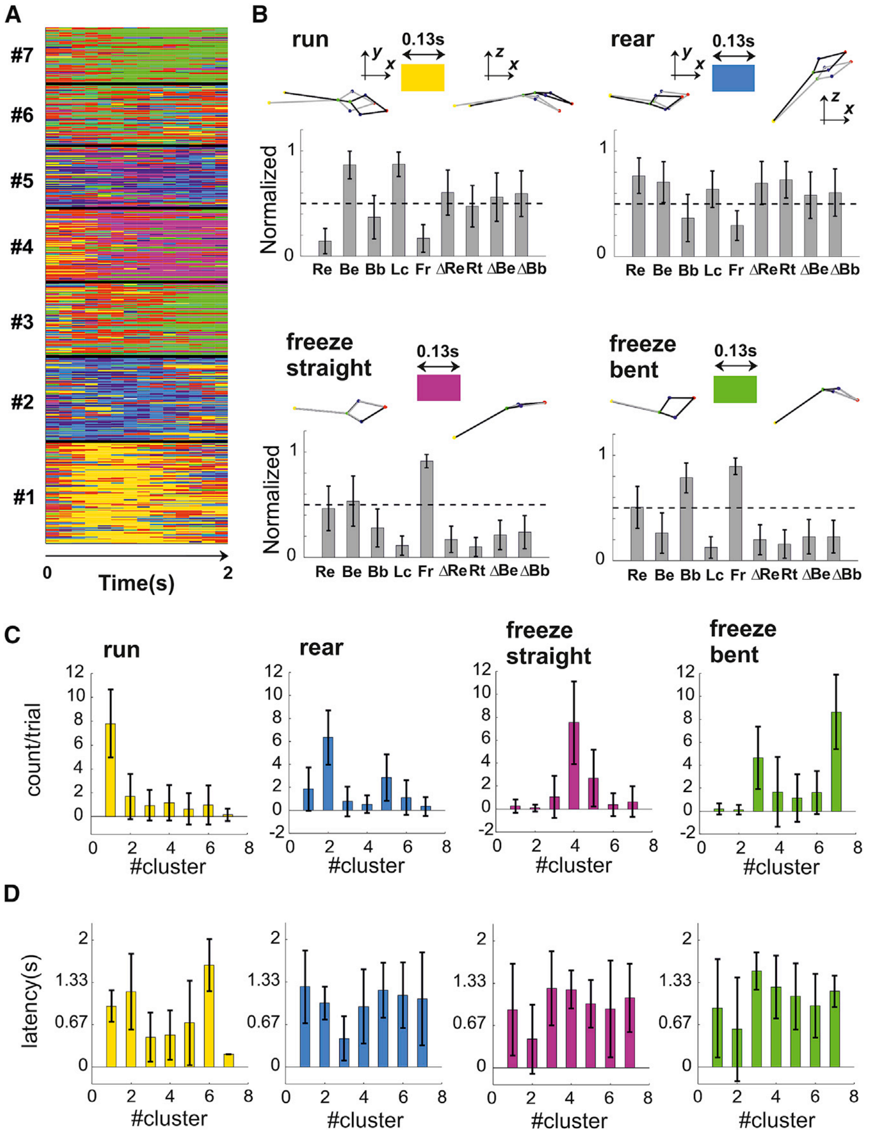

Figure 6. Distinct Behaviors Differ Both in Rate and Latency of Behavioral Primitives

(A) The primitives extracted from the response matrix are displayed for all trials (n = 8 primitives; duration = 0.133 s). Trials are partitioned into the 7 clusters as in Figure 5D.

(B) The mean ± SD of all measures of postures and movements are shown for four primitives (run, rear, freeze straight, and freeze bent). Individual representative samples of each primitive are shown as 3D body reconstructions at the top of each bar graph. For additional details, see Figures S7A–S7C.

(C) Frequency (mean ± SD) of each primitive across the 7 behavioral clusters shown in Figure 5D.

(D) Latency (mean ± SD) of each primitive across the 7 behavioral clusters shown in Figure 5D.

or the same set of primitives but expressed at different latencies from the stimulus onset. To better understand the composition of each behavior, we increased the temporal resolution of our behavioral analysis by subdividing the 2-s window into consecutive sub-second epochs. We then performed a clustering analysis across those sub-second epochs to identify the primitives. In order to select the number of primitives and their duration, we used a decoding approach. Thus, for each parameter combination, we fitted three stimulus-specific, variable-order Markov models (VMMs), one for each stimulus class (see STAR Methods section Analysis of Behavioral Primitives for details). Decoding performances were then evaluated on hold-out data by assigning each trial to the stimulus-specific VMMs associated with the highest likelihood. The VMMs cross-validated performances were optimal for primitive duration between 0.13 and 0.33 s (Figure S7A). Within this range, the best VMMs contained 6–8 primitives and exhibited maximum Markov order of 0 to 1 time steps (Figure S7B). We selected VMMs with 8 primitives of 0.13-s duration (Figures 6A and 6B), and we used them to compare, across the 7 behaviors, the rate and the latency of the primitives. For each stimulus, the distribution of primitives was significantly different from that observed during the spontaneous behavior preceding the stimulus (Figure S7C; p = 0, 0, and 0; Pearson's χ^2 test for flash, loom, and sound). For flash, the two most frequently occurring primitives defined the responses to clusters no. 1 and no. 2 in Figure 5D and represented, respectively, run and rear actions (Figure 6B). For loom and sound, the most frequent primitives were both expression of freezing but along different postures: with straight elongated body for loom (Figure 6B, freeze straight) and with hunched and left or right bent body for sound (Figure 6B, freeze bent). Both the latency and the rate of those primitives changed significantly across the 7 behaviors (Figure 6C; rate: p = 0, 0, 0, and 0; latency p = 0, 0, 0.0014, and 0; Kruskal-Wallis one-way ANOVA for run, rear, freeze straight, and freeze bent). These results indicate that both the composition and the timing of basic motor actions and postures varies in those behaviors.

The Mapping between Stimulus and Response Is Not Uniquely Defined by Observable Initial Conditions

From the results in Figure 5, a clear "one-to-many" mapping emerges, in which each stimulus can evoke multiple behavioral responses. Such multiplicity could be driven by several factors preceding the time of the stimulus onset and dynamically reconfiguring the mapping between stimulus and response: internal states of the animal that are independent from the stimuli and ongoing observable behaviors; variable postures and motor states that mechanically constrain the range of possible behavioral responses; and variable position of eyes and ears within the behavioral arena that modify the way the same stimulus is perceived across trials.

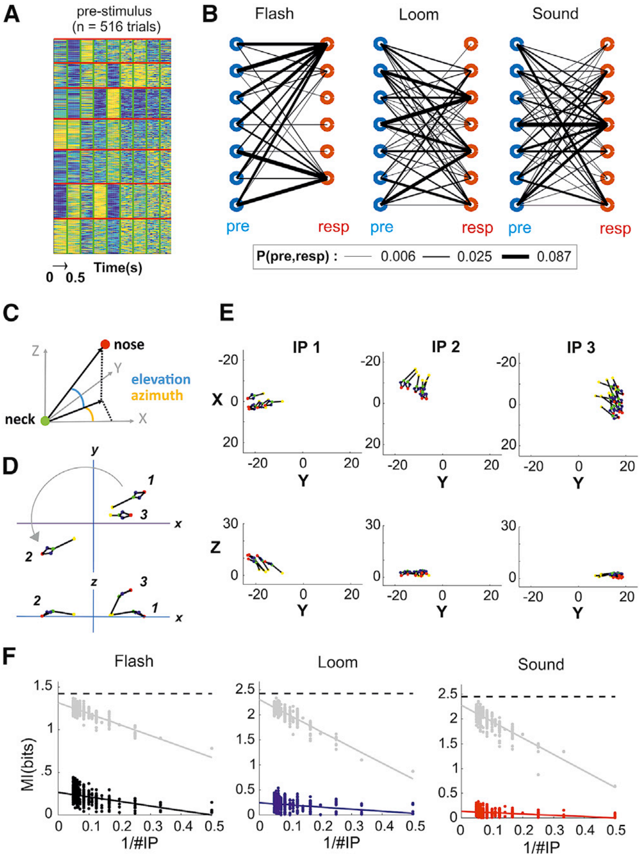

Figure 7. The Mapping between Stimulus and Response Is Not Uniquely Defined by Initial Conditions

(A) Matrix representing the concatenation of all the measures of posture and movements for the 0.5 s preceding the stimulus onset. Trials (n = 516) have been partitioned in 7 clusters to match the cardinality of response clustering shown in Figure 5D.

(B) Joint probability of pre-stimulus (blue circles) and response clusters (red circles) for flash, loom, and sound stimuli. The probability value is proportional to the width of the lines connecting pre-stimulus and response as shown in legend. For the analysis of robustness of such results as function of goodness of clustering, see Figures S7D–S7G.

(C) Head elevation is calculated as the vertical angle between nose and neck and head azimuth as the angle of the nose projection on the X-Y plane.

(D) Example of three initial positions. Position 2 is distant from position 1 along the X-Y coordinates but can be exactly superimposed to it by a single rotation along the Z axis. Position 3 is closer to position 1 along the X-Y coordinates, but in order to superimpose these two positions, a translation and two rotations are required.

(E) Example of three partitions of initial positions from the dataset; each pose represents an individual trial.

(F) MI is estimated as function of the inverse of the overall number of partitions (1/#IP) across 5 dimensions (head elevation and azimuth and head X,Y,Z coordinates). The dotted black lines indicate the entropy of the response clusters.

We first set out to explore the effect of ongoing posture and motor state (hereafter for simplicity referred to as ongoing activity). We tested the hypothesis that, given a particular stimulus, the ongoing activity uniquely defined the subsequent behavioral response. To this end, we first performed a clustering analysis on the epochs immediately preceding stimulus onset (duration = 0.5 s). Each cluster identified different ongoing activities, and the number of clusters was predefined and equal to 7 in order to match the cardinality of the response clusters (Figure 7A). If ongoing activities were to uniquely define the response, we would expect a "one-to-one" mapping. We found this not to be the case. Consistently with the one-to-many mapping previously described, each ongoing activity cluster led to multiple responses (Figure 7B). To quantify the dependence of response from ongoing activity we used MI. We found that ongoing activity could only account for a small fraction of the MI required to optimally predict the responses (14.92% flash, 7.2% loom, and 4.77% sound).

A caveat of this analysis lies in the fact that the multiplicity of responses might trivially arise from the hard boundaries imposed by the clustering procedure. Thus, high-dimensional points, representing either ongoing activities or responses, located near the boundaries between two or more clusters would still be assigned to one cluster only. To address the possibility that a one-to-many mapping simply arises from trials whose cluster membership is weakly defined, we developed a procedure to remove such trials (see STAR Methods section Clustering Refinement). By removing an increasing number of trials, the overall goodness of clustering increased both for ongoing activities and responses (Figures S7D and S7E). In this reduced dataset (293 trials out of 516), individual clusters of ongoing activities still led to multiple responses (Figures S7F and S7G) and only accounted for a small fraction of the MI required for correct prediction of the response cluster (11.92% flash, 9.01% loom, and 4.85% sound), indicating that the one-to-many mapping was robust to clustering errors.

We then set out to investigate the effect of the position of eyes and ears at the time of stimulus onset (hereafter simply referred to as initial position). We quantified initial positions by measuring 5 dimensions: head orientation (elevation and azimuth; Figure 7C) and the head X-Y-Z position. All these dimensions were calculated in allocentric coordinates in respect to the center of the arena (see STAR Methods section Estimating the Effects of Initial Positions). Because all our measures of movements and postures are instead expressed in egocentric coordinates, it is not clear how to connect these two coordinate systems. For example, it is possible that initial positions distant from each other in X-Y coordinates but well matched after a rotation around the Z axis would provide more (or less) similar responses than initial positions closer to each other in X-Y coordinates but with poor rotational symmetry (Figure 7D). In order to avoid any assumption about the mapping between egocentric responses and allocentric coordinates, we developed a systematic method to extrapolate the effect of initial positions on behavioral responses. Our method relies on the fact that, in the limit of an infinite number of partitions in the space of initial positions, a one-to-one mapping between initial positions and behavioral responses, if present, will always enable a correct prediction of the response cluster from the initial position. To test for this possibility, we systematically increased the number of partitions (see example partitions in Figure 7E), and each time, we calculated the MI between the initial positions and the response clusters (see black, blue, and red dots in Figure 7F). We then used linear extrapolation to estimate the MI in the limit of an infinite number of partitions clusters (see black, blue, and red lines in Figure 7F;

see STAR Methods section Estimating the Effects of Initial Positions). We found that initial positions only accounted for a minority of the MI required for correct prediction of the response cluster (18.76% flash, 10.06% loom, and 5.46% sound). Similar results were obtained after removal of 50% of the trials, for which the cluster membership for the responses was weakly defined (14.53% flash, 8.46% loom, and 8.99% sound). In principle, it possible that our linear extrapolation substantially underestimates the information conveyed by initial positions. However, when the order of the trials for initial positions and response clusters were separately re-organized to maximize their match, our extrapolation of the MI well captured the entropy of the response clusters (92.68%, 95%, and 93.03% of entropy for flash, loom, and sound; Figure 7F, gray dots and lines). This indicates that our extrapolation could capture a one-to-one mapping between initial positions and behavioral responses, but such mapping was not present in the data.

DISCUSSION

A fundamental goal of neuroscience is to link neural circuits to behaviors. Two unescapable tasks are essential prerequisites for approaching this problem: the generation of a detailed anatomical and physiological description of brain circuits—the neural repertoire—and the charting of all the relevant behaviors exhibited by the model organism of choice—the behavioral repertoire. Then, in order to uncover meaningful links, the resolutions of the neural and the behavior repertoires have to match, because a high resolution on one side cannot compensate for low resolution on the other [15].

In the last decade, enormous advances have been made in understanding functional and anatomical connectivity of the CNS [16–18]. Thanks to these techniques, a detailed sketching of the neural repertoire underlying sensory-guided defensive behaviors in the mouse is in process and substantial advances have been made in the last few years [6, 9, 19–22].

High-dimensional reconstruction of rodent behavior is now starting to catch up (see, e.g., [23, 24] for comprehensive reviews). Such reconstructions have been first developed for constrained situations (e.g., treadmill walk) and by applying physical markers to detect body landmarks [25]. More recently, machine learning [26–28] and deep learning [29–31] have allowed to obviate for the need to use physical markers. Alternative approaches have also been taken by using depth cameras [32] or by combining traditional video with head-mounted sensors to measure head movements [33] and even eye movements and pupil constriction [34]. In spite of these advancements, the behavioral repertoire for defensive behaviors has so far only been quantified by measuring changes in locomotion state.

The first aim of this work was to provide a higher resolution map of sensory-guided behaviors. To achieve this aim, we used four cameras that allowed us to triangulate 2D body landmarks and obtain a 3D reconstruction of the mouse body. The accuracy of such a reconstruction was substantially improved by training 3D SSM that we used to correct the 3D coordinates (Figure S2). Our approach is supervised in that it requires to pre-specify a set of body landmarks (nose, ears, neck base, body center, and tail base; see Figure 1A). Previous approaches to perform a mouse 3D reconstruction, realized by using a depth camera, took instead an unsupervised approach using all body points in the images followed by dimensionality reduction [32, 35]. The main advantage of our supervised approach relies on the fact that the poses are easier to interpret. For example, a mouse looking up can be easily described by a change in nose elevation in respect to the neck base. The main disadvantage is represented by the potential errors in 3D reconstruction arising from incorrect tracking of body landmarks. However, reconstruction errors can be minimized by using multiple camera views and SSMs, and this approach is easily scalable to any number of views.

Our first main finding was that the level of stimulus-response specificity provided by a high-dimensional description of mouse behavior is higher than the specificity measured with locomotion alone (Figures 3 and 4). This increase in specificity was particularly remarkable when comparing behavioral responses to a loud sound and a visual looming. It has been previously shown that both stimuli induce escape to a shelter or freeze when the shelter is not present [7, 36]. As a result, the responses to these stimuli have been considered equivalent and no attempts have been made to differentiate them. Here, we show that looming and sound responses can be discriminated with ∼78% accuracy (Figure 4). This result can be explained by the fact that a higher dimensional behavioral quantification revealed a larger number of distinct behaviors that are stimulus specific. Thus, for both looming and sound, the animals typically froze, but they did so according to two different postures: a straight, upward-looking pose for loom (Figure 3A and cluster no. 4 in Figures 5 and 6) and a hunched pose for sound often preceded by a body spin (Figure 3A and cluster no. 3 in Figures 5 and 6). Moreover, in several trials, a looming stimulus was more likely than sound to elicit rearing or short-lasting freeze in rearing position (clusters no. 2 and no. 5 in Figures 5 and 6).

In locomotion data, where this diversity was lost (Figure 5), specificity for looming and sound was substantially reduced (Figure 4). Linking the neural repertoire to the behavioral repertoire based on locomotion alone would indicate almost perfect convergence—different sensory processes ultimately lead to only one single action. Instead, by increasing the resolution of the behavioral repertoire, we were able to reject the convergence hypothesis, showing that behavioral outputs preserve a significant level of stimulus specificity.

For other pairs of stimuli, such as flash versus loom, locomotion alone granted a good level of discrimination (≈90% accuracy; Figure 4). A higher dimensional quantification of postures and movements did not provide substantial advantages in discriminating between such stimuli but enabled to better describe behavioral responses. Therefore, although locomotion data could well differentiate a response to a flash as opposed to a looming stimulus, a higher dimensional quantification could tell us whether the animal was rearing or running (clusters no. 1 and no. 2 in Figures 5 and 6).

Our second main finding was a one-to-many mapping between stimulus and response. Thus, a high-dimensional description revealed at least seven behavioral responses, and each stimulus could evoke at least three (Figure 5). The same analysis on locomotion data identified only two behaviors across all stimuli (Figures 5B and 5C). The reduced, essentially binary, mapping between stimulus and response is consistent with previous

results that employed locomotion as unique behavioral descriptor. In absence of shelter, a looming stimulation was shown to evoke either immediate freeze or escape followed by freeze [9]. When a shelter was present, a dark sweeping object typically evoked a freeze, but flight was also observed in a smaller number of trials [5]. Our higher dimensional descriptors provide a substantially enhanced picture of this phenomenon and indicate that the one-to-many mapping between stimulus and response occurs robustly across different sensory stimuli.

The overall figure of seven distinct behaviors represents a conservative estimate and reflects the criterion we used to define the granularity of our behavioral classification. Previous studies, aimed at providing an exhaustive description of spontaneous behaviors, identified ~60 distinct classes in the mouse [32] and ~100 in fruit fly [28]. The smaller set of behaviors identified in this study, although more tractable and still sufficient for capturing stimulus-response specificity, likely underestimates the repertoire of mouse defensive actions.

The one-to-many mapping we described could not be trivially explained by different initial conditions, i.e., by the variety of postures and motor states or by the position of eyes and ears at the time of stimulus presentation (Figure 7). This is consistent with recent results in *Drosophila*, where ongoing behavior had statistically significant, but not deterministic, effects on future behaviors [37] and on responses to optogenetic stimulation of descending neurons [38]. Therefore, at least to some extent, the one-to-many mapping reflects stimulus-independent variability in the internal state of the animal that generates diversity in the behavioral output. Variability in the internal states could take many forms, ranging from noise in the neuronal encoding of the stimuli along the visual and auditory pathways [39] to fluctuating levels of arousal [40, 41] or anxiety [42], and further studies will be required to discriminate among those contributions. The high level of functional degeneracy in neuronal networks (see, e.g., [43–45]) provides the suitable substrate for the observed behavioral diversity. The presence of functional degeneracy is consistent with recent studies reporting that the expression of defensive responses can be affected by activation of multiple neuronal pathways [9, 10, 46–50]. However, our current understanding of the anatomical and functional substrates of this diversity is still insufficient and limited to the locomotion phenotype. We believe that further investigations of such substrates, matched with a more-detailed description of defensive behaviors, represent an important avenue for future studies.

STAR★METHODS

Detailed methods are provided in the online version of this paper and include the following:

- KEY RESOURCES TABLE
- RESOURCE AVAILABILITY
  - ○ Lead Contact
  - ○ Materials Availability
  - ○ Data and Code Availability
- EXPERIMENTAL MODEL AND SUBJECT DETAILS
  - ○ Animals
  - ○ Ethical Statement
- METHOD DETAILS
  - ○ Behavioral Experiments
  - ○ Visual and Auditory Stimuli
  - ○ Experimental Set-Up
- QUANTIFICATION AND STATISTICAL ANALYSIS
  - ○ Reconstruction of 3D poses
  - ○ Validation of the 3D reconstruction
  - ○ Interpretation of the eigenposes
  - ○ Normalization of the behavioral measures
  - ○ Validation of the postural and movement measures
  - ○ Response Divergence
  - ○ Rank estimation
  - ○ Stimulus-response specificity
  - ○ Decoding Analysis
  - ○ Clustering and Information Analysis
  - ○ Analysis of Behavioral Primitives
  - ○ Clustering Refinement
  - ○ Estimating the Effects of Initial Positions

SUPPLEMENTAL INFORMATION

Supplemental Information can be found online at https://doi.org/10.1016/j.cub.2020.09.007.

ACKNOWLEDGMENTS

This study was funded by a David Sainsbury Fellowship from National Centre for Replacement, Refinement and Reduction of Animals in Research (NC3Rs) to R.S. (NC/P001505/1), by a Medical Research Council grant to R.J.L. (MR/N012992/1), and by a Fight for Sight Fellowship to N.M. (5047/5048).

AUTHOR CONTRIBUTIONS

Conceptualization, R.S. and R.J.L.; Methodology, R.S., A.G.Z., and T.F.C.; Formal Analysis, R.S., A.A., and A.G.Z.; Investigation, R.S. and N.M.; Writing, R.S., N.M., A.E.A., and R.J.L.; Funding Acquisition, R.S. and R.J.L.

DECLARATION OF INTERESTS

The authors declare no competing interests.

Received: March 13, 2020
Revised: July 6, 2020
Accepted: September 3, 2020
Published: October 1, 2020

REFERENCES

1. Cooke, S.F., Komorowski, R.W., Kaplan, E.S., Gavornik, J.P., and Bear, M.F. (2015). Erratum: Visual recognition memory, manifested as long-term habituation, requires synaptic plasticity in V1. Nat. Neurosci. *18*, 926.
2. Smeds, L., Takeshita, D., Turunen, T., Tiihonen, J., Westö, J., Martyniuk, N., Seppänen, A., and Ala-Laurila, P. (2019). Paradoxical rules of spike train decoding revealed at the sensitivity limit of vision. Neuron *104*, 576–587.e11.
3. Hoy, J.L., Bishop, H.I., and Niell, C.M. (2019). Defined cell types in superior colliculus make distinct contributions to prey capture behavior in the mouse. Curr. Biol. *29*, 4130–4138.e5.
4. Hoy, J.L., Yavorska, I., Wehr, M., and Niell, C.M. (2016). Vision drives accurate approach behavior during prey capture in laboratory mice. Curr. Biol. *26*, 3046–3052.
5. De Franceschi, G., Vivattanasarn, T., Saleem, A.B., and Solomon, S.G. (2016). Vision guides selection of freeze or flight defense strategies in mice. Curr. Biol. *26*, 2150–2154.

CellPress
OPEN ACCESS

6. Evans, D.A., Stempel, A.V., Vale, R., Ruehle, S., Lefler, Y., and Branco, T. (2018). A synaptic threshold mechanism for computing escape decisions. Nature *558*, 590–594.
7. Storchi, R., Rodgers, J., Gracey, M., Martial, F.P., Wynne, J., Ryan, S., Twining, C.J., Cootes, T.F., Killick, R., and Lucas, R.J. (2019). Measuring vision using innate behaviours in mice with intact and impaired retina function. Sci. Rep. *9*, 10396.
8. Yilmaz, M., and Meister, M. (2013). Rapid innate defensive responses of mice to looming visual stimuli. Curr. Biol. *23*, 2011–2015.
9. Shang, C., Chen, Z., Liu, A., Li, Y., Zhang, J., Qu, B., Yan, F., Zhang, Y., Liu, W., Liu, Z., et al. (2018). Divergent midbrain circuits orchestrate escape and freezing responses to looming stimuli in mice. Nat. Commun. *9*, 1232.
10. Salay, L.D., Ishiko, N., and Huberman, A.D. (2018). A midline thalamic circuit determines reactions to visual threat. Nature *557*, 183–189.
11. Redgrave, P., Dean, P., Souki, W., and Lewis, G. (1981). Gnawing and changes in reactivity produced by microinjections of picrotoxin into the superior colliculus of rats. Psychopharmacology (Berl.) *75*, 198–203.
12. Dean, P., Redgrave, P., and Westby, G.W. (1989). Event or emergency? Two response systems in the mammalian superior colliculus. Trends Neurosci. *12*, 137–147.
13. Koch, M. (1999). The neurobiology of startle. Prog. Neurobiol. *59*, 107–128.
14. Owen, A.B., and Perry, P.O. (2009). Bi-cross-validation of the SVD and the nonnegative matrix factorization. Ann. Appl. Stat. *3*, 564–594.
15. Krakauer, J.W., Ghazanfar, A.A., Gomez-Marin, A., MacIver, M.A., and Poeppel, D. (2017). Neuroscience needs behavior: correcting a reductionist bias. Neuron *93*, 480–490.
16. Bernstein, J.G., and Boyden, E.S. (2011). Optogenetic tools for analyzing the neural circuits of behavior. Trends Cogn. Sci. *15*, 592–600.
17. Oh, S.W., Harris, J.A., Ng, L., Winslow, B., Cain, N., Mihalas, S., Wang, Q., Lau, C., Kuan, L., Henry, A.M., et al. (2014). A mesoscale connectome of the mouse brain. Nature *508*, 207–214.
18. Erö, C., Gewaltig, M.O., Keller, D., and Markram, H. (2018). A cell atlas for the mouse brain. Front. Neuroinform. *12*, 84.
19. Reinhard, K., Li, C., Do, Q., Burke, E.G., Heyndrickx, S., and Farrow, K. (2019). A projection specific logic to sampling visual inputs in mouse superior colliculus. eLife *8*, e50697.
20. Shang, C., Liu, Z., Chen, Z., Shi, Y., Wang, Q., Liu, S., Li, D., and Cao, P. (2015). BRAIN CIRCUITS. A parvalbumin-positive excitatory visual pathway to trigger fear responses in mice. Science *348*, 1472–1477.
21. Tovote, P., Esposito, M.S., Botta, P., Chaudun, F., Fadok, J.P., Markovic, M., Wolff, S.B.E., Ramakrishnan, C., Fenno, L., Deisseroth, K., et al. (2016). Midbrain circuits for defensive behaviour. Nature *534*, 206–212.
22. Wei, P., Liu, N., Zhang, Z., Liu, X., Tang, Y., He, X., Wu, B., Zhou, Z., Liu, Y., Li, J., et al. (2015). Processing of visually evoked innate fear by a non-canonical thalamic pathway. Nat. Commun. *6*, 6756.
23. Datta, S.R., Anderson, D.J., Branson, K., Perona, P., and Leifer, A. (2019). Computational neuroethology: a call to action. Neuron *104*, 11–24.
24. Mathis, M.W., and Mathis, A. (2020). Deep learning tools for the measurement of animal behavior in neuroscience. Curr. Opin. Neurobiol. *60*, 1–11.
25. Courtine, G., Gerasimenko, Y., van den Brand, R., Yew, A., Musienko, P., Zhong, H., Song, B., Ao, Y., Ichiyama, R.M., Lavrov, I., et al. (2009). Transformation of nonfunctional spinal circuits into functional states after the loss of brain input. Nat. Neurosci. *12*, 1333–1342.
26. Machado, A.S., Darmohray, D.M., Fayad, J., Marques, H.G., and Carey, M.R. (2015). A quantitative framework for whole-body coordination reveals specific deficits in freely walking ataxic mice. eLife *4*, e07892.
27. Petersen, R.S., Colins Rodriguez, A., Evans, M.H., Campagner, D., and Loft, M.S.E. (2020). A system for tracking whisker kinematics and whisker shape in three dimensions. PLoS Comput. Biol. *16*, e1007402.
28. Berman, G.J., Choi, D.M., Bialek, W., and Shaevitz, J.W. (2014). Mapping the stereotyped behaviour of freely moving fruit flies. J. R. Soc. Interface *11*, 20140672.
29. Mathis, A., Mamidanna, P., Cury, K.M., Abe, T., Murthy, V.N., Mathis, M.W., and Bethge, M. (2018). DeepLabCut: markerless pose estimation of user-defined body parts with deep learning. Nat. Neurosci. *21*, 1281–1289.
30. Pereira, T.D., Aldarondo, D.E., Willmore, L., Kislin, M., Wang, S.S., Murthy, M., and Shaevitz, J.W. (2019). Fast animal pose estimation using deep neural networks. Nat. Methods *16*, 117–125.
31. Graving, J.M., Chae, D., Naik, H., Li, L., Koger, B., Costelloe, B.R., and Couzin, I.D. (2019). DeepPoseKit, a software toolkit for fast and robust animal pose estimation using deep learning. eLife *8*, e47994.
32. Wiltschko, A.B., Johnson, M.J., Iurilli, G., Peterson, R.E., Katon, J.M., Pashkovski, S.L., Abraira, V.E., Adams, R.P., and Datta, S.R. (2015). Mapping sub-second structure in mouse behavior. Neuron *88*, 1121–1135.
33. Klaus, A., Martins, G.J., Paixao, V.B., Zhou, P., Paninski, L., and Costa, R.M. (2017). The spatiotemporal organization of the striatum encodes action space. Neuron *95*, 1171–1180.e7.
34. Meyer, A.F., Poort, J., O'Keefe, J., Sahani, M., and Linden, J.F. (2018). A head-mounted camera system integrates detailed behavioral monitoring with multichannel electrophysiology in freely moving mice. Neuron *100*, 46–60.e7.
35. Markowitz, J.E., Gillis, W.F., Beron, C.C., Neufeld, S.Q., Robertson, K., Bhagat, N.D., Peterson, R.E., Peterson, E., Hyun, M., Linderman, S.W., et al. (2018). The striatum organizes 3D behavior via moment-to-moment action selection. Cell *174*, 44–58.
36. Vale, R., Evans, D.A., and Branco, T. (2017). Rapid spatial learning controls instinctive defensive behavior in mice. Curr. Biol. *27*, 1342–1349.
37. Berman, G.J., Bialek, W., and Shaevitz, J.W. (2016). Predictability and hierarchy in Drosophila behavior. Proc. Natl. Acad. Sci. USA *113*, 11943–11948.
38. Cande, J., Namiki, S., Qiu, J., Korff, W., Card, G.M., Shaevitz, J.W., Stern, D.L., and Berman, G.J. (2018). Optogenetic dissection of descending behavioral control in *Drosophila*. eLife *7*, e34275.
39. Faisal, A.A., Selen, L.P., and Wolpert, D.M. (2008). Noise in the nervous system. Nat. Rev. Neurosci. *9*, 292–303.
40. Schröder, S., Steinmetz, N.A., Krumin, M., Pachitariu, M., Rizzi, M., Lagnado, L., Harris, K.D., and Carandini, M. (2020). Arousal modulates retinal output. Neuron *107*, 487–495.e9.
41. Liang, L., Fratzl, A., Reggiani, J.D.S., El Mansour, O., Chen, C., and Andermann, M.L. (2020). Retinal inputs to the thalamus are selectively gated by arousal. Curr. Biol. Published online August 11, 2020. https://doi.org/10.1016/j.cub.2020.07.065.
42. Gründemann, J., Bitterman, Y., Lu, T., Krabbe, S., Grewe, B.F., Schnitzer, M.J., and Lüthi, A. (2019). Amygdala ensembles encode behavioral states. Science *364*, eaav8736.
43. Drion, G., O'Leary, T., and Marder, E. (2015). Ion channel degeneracy enables robust and tunable neuronal firing rates. Proc. Natl. Acad. Sci. USA *112*, E5361–E5370.
44. Edelman, G.M., and Gally, J.A. (2001). Degeneracy and complexity in biological systems. Proc. Natl. Acad. Sci. USA *98*, 13763–13768.
45. Tononi, G., Sporns, O., and Edelman, G.M. (1999). Measures of degeneracy and redundancy in biological networks. Proc. Natl. Acad. Sci. USA *96*, 3257–3262.
46. Barbano, M.F., Wang, H.L., Zhang, S., Miranda-Barrientos, J., Estrin, D.J., Figueroa-González, A., Liu, B., Barker, D.J., and Morales, M. (2020). VTA glutamatergic neurons mediate innate defensive behaviors. Neuron *107*, 368–382.e8.
47. Huang, L., Yuan, T., Tan, M., Xi, Y., Hu, Y., Tao, Q., Zhao, Z., Zheng, J., Han, Y., Xu, F., et al. (2017). A retinoraphe projection regulates serotonergic activity and looming-evoked defensive behaviour. Nat. Commun. *8*, 14908.
48. Liang, F., Xiong, X.R., Zingg, B., Ji, X.Y., Zhang, L.I., and Tao, H.W. (2015). Sensory cortical control of a visually induced arrest behavior via corticotectal projections. Neuron *86*, 755–767.

49. Chou, X.L., Wang, X., Zhang, Z.G., Shen, L., Zingg, B., Huang, J., Zhong, W., Mesik, L., Zhang, L.I., and Tao, H.W. (2018). Inhibitory gain modulation of defense behaviors by zona incerta. Nat. Commun. *9*, 1151.

50. Li, L., Feng, X., Zhou, Z., Zhang, H., Shi, Q., Lei, Z., Shen, P., Yang, Q., Zhao, B., Chen, S., et al. (2018). Stress accelerates defensive responses to looming in mice and involves a locus coeruleus-superior colliculus projection. Curr. Biol. *28*, 859–871.e5.

51. Hurst, J.L., and West, R.S. (2010). Taming anxiety in laboratory mice. Nat. Methods *7*, 825–826.

52. Peirce, J.W. (2007). PsychoPy–Psychophysics software in Python. J. Neurosci. Methods *162*, 8–13.

53. Govardovskii, V.I., Fyhrquist, N., Reuter, T., Kuzmin, D.G., and Donner, K. (2000). In search of the visual pigment template. Vis. Neurosci. *17*, 509–528.

54. Jacobs, G.H., and Williams, G.A. (2007). Contributions of the mouse UV photopigment to the ERG and to vision. Doc. Ophthalmol. *115*, 137–144.

55. Hartley, R.I.Z.A. (2004). Multiple View Geometry in Computer Vision, Second Edition (Cambridge University).

56. Cootes, T.F., Taylor, C.J., Cooper, D.H., and Graham, J. (1995). Active shape models - their training and application. Comput. Vis. Image Underst. *61*, 38–59.

57. Arthur, D., and Vassilvitskii, S. (2007). k-means++: the advantages of careful seeding. Proceedings of the Eighteenth Annual ACM-SIAM Symposium on Discrete Algorithms (Society for Industrial and Applied Mathematics), pp. 1027–1035.

58. Storchi, R., Zippo, A.G., Caramenti, G.C., Valente, M., and Biella, G.E. (2012). Predicting spike occurrence and neuronal responsiveness from LFPs in primary somatosensory cortex. PLoS ONE *7*, e35850.

59. Storchi, R., Bale, M.R., Biella, G.E., and Petersen, R.S. (2012). Comparison of latency and rate coding for the direction of whisker deflection in the subcortical somatosensory pathway. J. Neurophysiol. *108*, 1810–1821.

60. Milosavljevic, N., Storchi, R., Eleftheriou, C.G., Colins, A., Petersen, R.S., and Lucas, R.J. (2018). Photoreceptive retinal ganglion cells control the information rate of the optic nerve. Proc. Natl. Acad. Sci. USA *115*, E11817–E11826.

61. Strong, S.P., Koberle, R., de Ruyter van Steveninck, R.R., and Bialek, W. (1998). Entropy and information in neural spike trains. Phys. Rev. Lett. *80*, 197–200.

62. Rissanen, J. (1983). A universal data compression system. IEEE Trans. Inf. Theory *29*, 656–664.

63. Begleiter, R., El-Yaniv, R., and Yona, G. (2004). On prediction using variable order Markov models. J. Artif. Intell. Res. *22*, 385–421.

64. Cleary, J., and Witten, I. (1984). Data compression using adaptive coding and partial string matching. IEEE Trans. Commun. *32*, 396–402.

STAR★METHODS

KEY RESOURCES TABLE

REAGENT or RESOURCE	SOURCE	IDENTIFIER
Experimental Models: Organisms/Strains		
C576BL/6J	University of Manchester	RRID:MGI:5811150
Software and Algorithms		
MATLAB R2017a	The Mathworks	https://www.mathworks.com/products/matlab.html
Python	Python Software Foundation	https://www.python.org/
PsychoPy	Jonathan Peirce	http://www.psychopy.org/
Arduino	Arduino	http://www.arduino.cc
FlyCapture2	FLIR	https://www.flir.co.uk/products/flycapture-sdk/
Deposited Data		
Full 3D dataset of mouse poses	University of Manchester	https://github.com/RStorchi/HighDimDefenseBehaviours

RESOURCE AVAILABILITY

Lead Contact

Further information and requests for resources, reagents or raw data should be directed to and will be fulfilled by the Lead Contact, Riccardo Storchi (riccardo.storchi@manchester.ac.uk)

Materials Availability

This study did not generate new unique reagents.

Data and Code Availability

Data and source codes are available at https://github.com/RStorchi/HighDimDefenseBehaviours

EXPERIMENTAL MODEL AND SUBJECT DETAILS

Animals

In this study we used C57BL/6 mice (n = 29, all male) obtained from obtained from the Biological Services facility at University of Manchester. All mice were stored in cages of 3 individuals and were provided with food and water *ad libitum*. Mice were kept on a 12:12 light dark cycle.

Ethical Statement

Experiments were conducted in accordance with the Animals, Scientific Procedures Act of 1986 (United Kingdom) and approved by the University of Manchester ethical review committee.

METHOD DETAILS

Behavioral Experiments

The animals were recorded in a square open field arena (dimensions: 30cm x 30 cm; Figures S1A and S1B). Experiments were conducted at Zeitgeber time 6 or 18 (respectively n = 14 and 15 animals). During transfer between the cage and the behavioral arena we used the tube handling procedure instead of tail picking, as prescribed in [51], in order to minimize stress and reduce variability across animals. After transferring to the behavioral arena the animals were allowed 10 minutes to habituate to the environment before starting the experiment. Auditory white noise background at 64 dB(C) and background illumination ($4.08*10^{10}$, $1.65*10^{13}$, $1.94*10^{13}$ and $2.96*10^{13}$ photon/cm^2/s respectively S-cone opsin, Melanopsin, Rhodopsin and M-cone opsin) were delivered throughout habituation and testing. In each experiment we delivered 6 blocks of stimuli where each block was constituted by a flash, a looming and a sound. The order of the stimuli was independently randomized within each block. The inter-stimulus-interval was fixed at 70 s.

Visual and Auditory Stimuli

The flash stimulus provided diffuse excitation of all photoreceptors (S-cone opsin: $4.43*10^{12}$ photon/cm^2/s; Melanopsin: $2.49*10^{15}$ photon/cm^2/s; Rhodopsin: $1.98*10^{15}$ photon/cm^2/s; M-cone opsin: $7.09*10^{14}$ photon/cm^2/s). As looming stimulus we used two variants: a "standard" black looming (87% Michelson Contrast; looming speed = 66deg/s) and a modified looming where the black disc

was replaced by a disc with a grating pattern (Spatial Frequency = 0.068 cycles/degree; Michelson Contrast: 35% for white versus gray, 87% for gray versus black, 94% for white versus black; looming speed = 66deg/s). As auditory stimuli we used either a pure tone (C6 at 102 dB(C)) or a white noise (at 89 dB(C)) both presented for 1 s. The selection of looming and sound variants was randomly generated at each trial.

Experimental Set-Up

The animals were recorded with 4 programmable cameras (Chamaleon 3 from Point Grey; frame rate = 15Hz). The camera lenses were covered with infrared cut-on filters (Edmund Optics) and fed with constant infrared light. The experiments were controlled by using Psychopy (version 1.82.01) [52]. Frame acquisition was synchronized with the projected images and across cameras by a common electrical trigger delivered by an Arduino Uno board (arduino.cc) controlled by Psychopy through a serial interface (pyserial). Trigger control was enabled on Chamaleon 3 cameras through FlyCapture2 software (from Point Grey). All videos were encoded as M-JPEG from RGB 1280 (W) x 1040 (H) images. For tracking RGB images were converted to grayscale.

In order to deliver the flash stimulation we used two LEDs mounted inside the arena (model LZ4-00B208, LED engin; controlled by T-Cube drivers, Thorlabs). The auditory stimuli were provided by two speakers positioned outside the arena. Background illumination and the looming stimuli were delivered by a projector onto a rear projection screen mounted at the top of the arena. Calculation of retinal irradiance for each photoreceptor was based on Govardovskii templates [53] and lens correction functions [54].

QUANTIFICATION AND STATISTICAL ANALYSIS

Reconstruction of 3D poses

Three dimensional reconstruction of the mouse body was based on simultaneously tracked body landmarks from four the cameras (Figures S1A and S1B). The four camera system was calibrated using the Direct Linear Transform algorithm [55] before data collection by using Lego® objects of known dimensions (Figures S1C–S1F). The reconstruction error after triangulation was 0.153 ± 0.0884SD cm. For source codes and a detailed description of the calibration process see online material (https://github.com/RStorchi/HighDimDefenseBehaviours/tree/master/3Dcalibration).

After data collection body landmarks were detected independently for each camera by using DeepLabCut software [29]. We used $n = 5$ body landmarks: the nose-tip, the left and right ears, the neck base and the tail base (as shown Figure 1A). When the likelihood of a landmark was higher than 0.5 the landmark was considered valid. Valid landmarks were then used to estimate the 3D coordinates of the body points using least square triangulation. The result of this initial 3D reconstruction was saved as raw reconstruction (Figure 1B, Raw).

The raw reconstruction contained outlier poses caused by incorrect or missing landmark detections (typically occurring when the relevant body parts were occluded). To correct those outliers we developed a method that automatically identifies correctly reconstructed body points and uses the knowledge of the geometrical relations between all points to re-estimate the incorrectly reconstructed (or missing) points. Knowledge of these geometrical relations was provided by a Statistical Shape Model (SSM).

We first estimated a statistical shape model (SSM) of the mouse body based on $n = 5$ body points [56]. This was achieved by using a set of 400 poses, each represented by a $n \times 3$ matrix $\boldsymbol{X}_{train}$ whose correct 3D reconstruction was manually assessed. During manual assessment the coordinate of each body landmark across the four cameras was evaluated by a human observer. When all landmark location (n = 20, 5 landmarks for each of the 4 cameras) were approved the associated 3D pose was labeled as correct. Each training pose $\boldsymbol{X}_{train}$ was then aligned to a reference pose using Partial Procrustes Superimposition (PPS) and the mean pose $\overline{\boldsymbol{X}}$ calculated. This algorithm estimates the 3×3 rotation matrix $\boldsymbol{R}$ and the $n \times 3$ translation $\boldsymbol{T}$ matrix that minimize the distance $\overline{\boldsymbol{X}} - (\boldsymbol{X}_{train}\boldsymbol{R} + \boldsymbol{T})_F$ calculated by using the Frobenius norm. A principal component analysis was then performed on the aligned poses to obtain a set of eigenposes $\mathbf{P}$ and eigenvalues λ. The first $p = 3$ eigenposes were sufficient to explain 90.37% of the variance associated with shape changes in our training set (42.68%, 30.85% and 16.84% respectively). Based on those eigenposes the SSM model enabled to express any aligned pose $\boldsymbol{X}$ as

$$\boldsymbol{X} = \overline{\boldsymbol{X}} + \sum_{i}^{p} b_i \boldsymbol{P}_i \qquad \text{(Equation 3)}$$

where b_i represent the shape parameters. To identify outlier poses each pose $\boldsymbol{X}$ was first aligned to the mean pose $\overline{\boldsymbol{X}}$ and shape parameters were estimated. A pose was labeled as incorrect when either the Euclidean distance between $\overline{\boldsymbol{X}}$ and $\boldsymbol{X}$ or any of the shape parameters exceeded pre-set thresholds.

Outlier poses could be corrected if only 1-2 body points were incorrectly reconstructed by using the remaining body points and the trained SSM. Correctly reconstructed body points, represented by the $(n-2) \times 3$ matrix $\mathbf{X}_{subset}$, were identified as the subset of points, out of all possible $(n-2)$ subsets, that minimized the distance $\overline{\boldsymbol{X}}_{subset} \boldsymbol{R}_{subset} + \boldsymbol{T}_{subset} - \boldsymbol{X}_{subset\,F}$. Here the matrices $\boldsymbol{R}_{subset}$ and $\boldsymbol{T}_{subset}$ were obtained by aligning the corresponding body points of the reference pose, $\overline{\boldsymbol{X}}_{subset}$, to the selected $(n-2) \times 3$ matrix $\boldsymbol{X}_{subset}$. The shape parameters b_i were treated and missing data and re-estimated by applying Piecewise Cubic Hermite Interpolation on the shape parameter time series. The corrected pose $\boldsymbol{X}$ was then re-estimated as $\boldsymbol{X} = (\overline{\boldsymbol{X}} + \sum_{i=1}^{p} b_i \boldsymbol{P}_i) \boldsymbol{R}_{subset} + \boldsymbol{T}_{subset}$.

These preliminary stages enabled to replace gross outliers in the raw 3D reconstruction. We then used all poses $\boldsymbol{X}$ and associated shape parameters as input for an optimization procedure aimed at obtaining a refined 3D reconstruction by minimizing the following cost function:

$$C(\boldsymbol{b},\boldsymbol{R},\boldsymbol{T}) = \overline{\boldsymbol{X}} - \left(\boldsymbol{X} + \sum_{i=1}^{p} b_i \boldsymbol{P}_i\right)\boldsymbol{R} + \boldsymbol{T}_F + \alpha \sum_{i=1}^{N_p} \frac{b_i^2}{\lambda_i} \qquad \text{(Equation 4)}$$

where the right-hand side of the *Equation 3* represents a regularization factor to penalize for excessive changes in body shape. The value for the regularization parameter α, set at 0.001, was determined by first applying this cost function to a simulated dataset. For all further analyses the time series of each element of $\boldsymbol{b}$, $\boldsymbol{R}$ and $\boldsymbol{T}$ were smoothed using the kernel w = [0.2 0.6 0.2]. After smoothing each rotation matrix $\boldsymbol{R}(t)$ was renormalized by using Singular Value Decomposition.

Following this reconstruction procedure the mouse pose at any given frame t was defined by shape parameters $\boldsymbol{b}(t)$ and rigid transformations $\boldsymbol{R}(t)$ and $\boldsymbol{T}(t)$ as reported in *Equation 1*. The final 3D poses were defined as refined reconstruction (Figure 1B, Refined). A dynamic visualization of the refined reconstruction can be found in Video S1. All 3D data and source codes for estimating SSM and the refined reconstruction can be found here:

https://github.com/RStorchi/HighDimDefenseBehaviours/tree/master/3Dreconstruction

Validation of the 3D reconstruction

In order to compare raw and refined poses we first quantified the number of outliers. A pose was defined as outlier when, once aligned with the reference pose, its Euclidean distance from the reference in a 15 dimensional space (5 body points along the X,Y,Z axes) was larger than 5cm. For the raw and refined poses we detected respectively %3.31 (1037/31320) and 1.26% (395/31320) outliers (Figures S2A and S2B). In the raw 3D reconstruction the outliers were widespread across 178 trials while in the refined 3D reconstruction the outliers were concentrated in 7 trials that were then removed for all the subsequent analyses. Among inlier poses the distance from reference pose was only slightly reduced (Figure S2C, inset). However for the refined inlier poses the distance from the reference pose was fully explained by only 3 components while 9 components were required for the raw inlier poses (Figure S2D). The low dimensional variability associated with the refined inlier poses reflects the constraints imposed by the SSM (via the 3 eigenposes) while the high dimensional variability associated with the raw inlier poses reflects the effect of high dimensional noise. Such low and high dimensional variability can be clearly observed for the whole dataset of inlier poses in Figure S2E.

Interpretation of the eigenposes

The SSM enabled to identify a set of eigenposes that captured coordinated changes in the 3D shape of the animal body encompassing all the five body landmarks (see Equation 2). To gain more intuitive insights about what type of shape changes were captured by each eigenpose it is useful to visualize those changes. We did so by creating a video (Video S2) where we applied a sinusoidal change to individual shape parameters in *Equation 3*. In this way, at any given time t and for the ith eigenpose, the mouse body could be described as $\boldsymbol{X}(\boldsymbol{t}) = \overline{\boldsymbol{X}} + \lambda_i\sqrt{6}\sin(2\pi t)\cdot\boldsymbol{P}_i$. By looking at the video it is apparent that each eigenpose captures coordinated changes in the distances between body landmarks and angles between head and body. To quantify those changes as function of each eigenpose we selected, based on the video inspection, a set of four measures: nose-tail distance, neck-tail distance and head-to-body angles on the XY and the YZ planes. We found that the first eigenpose best correlated with nose-tail distance and head-to-body on the YZ plane indicating that this eigenpose captures different levels of body elongation (Figures S3A and S3D). The second eigenpose best correlated with head-to-body on the XY plane thus capturing left-right bending (Figure S3B). The third eigenpose correlated best with neck-tail distance indicating again a change in body elongation (Figure S3C).

Normalization of the behavioral measures

The full set of posture and movement measures was calculated from the refined 3D reconstruction as analytically described in Figure 1D. Each measure was then quantile normalized in the range [0, 1]. First all the values of each measure (n = #time points x #trials = 320 × 516 = 165120) were ranked from low to high. Then, according to its rank, each value was assigned to an interval. Each interval contained the same number of values. The interval containing the lowest values was assigned to 0 and the interval containing the lowest value was assigned to 1. All intermediate intervals were linearly spaced in the range (0,1). Finally the values were converted to their interval number.

Validation of the postural and movement measures

In order to validate the measures of postures and movements (Figure 1C) we compared such measures with a manually annotated set. The human observer (AA) watched the behavioral videos and annotated the start and end timing of each action across a subset of data (18 trials from 24 mice, 18 trials/mouse). We focused on four annotated actions: "Walk," 'Turn," "Freeze" and "Rear." The action "Turn" included left/right bending of the body as well as full body rotations around its barycenter. The action "Rear" included both climbing up walls and standing on hind legs without touching the walls. All annotated actions lasted on average less than 1 s ("Walk": 0.71 s ± 0.49 s, n = 473; "Turn": 0.68 s ± 0.42 s, n = 214; "Rear": 0.88 s ± 0.78 s, n = 505; mean ± SD) except "Freeze" (1.12 s ± 0.70 s, n = 371; mean ± SD).

Overall the automatic measures of Locomotion, Body Rotation, Freeze and Rearing (Figure 1C) were well matched with manual annotations while also providing additional information about changes in body shape. Thus "Walk" was associated with the largest increase in Locomotion (Figure S4A, right panel) as well as an increase in Body Elongation and decrease in Rearing and Body Bending (Figure S4A, left panel). "Turn" was associated with the largest increase in Body Rotation and Body Bending (Figure S4B). "Freeze" was associated with the largest increase in our measure of Freeze and the largest decrease in Locomotion (Figure S4C, right pane). "Rear" was associated with the largest increase in our measure of Rearing and high sustained Body Elongation (Figure S4D, left pane).

Response Divergence

We first calculated the Euclidean distance D between average time series obtained from two stimuli. This measure was then normalized by the average distance D_{sh} obtained by randomly shuffling across trials the association between stimulus and response (n = 1000 shuffles). Finally response divergence was calculated as $((D - \langle D\_sh \rangle))/\langle D\_sh \rangle$. To test for significance we used a shuffle test. We counted the number of times D was larger than D_{sh} and identified response divergence as significant when $D > D_{sh}$ in more than 95% of the shuffle repeats.

Rank estimation

For rank estimation we used the Bi-Cross Validation method proposed by Owen and Perry [14]. The $m \times n$ response matrix $\boldsymbol{X}$ is partitioned into four submatrices $\boldsymbol{A}$, $\boldsymbol{B}$, $\boldsymbol{C}$, $\boldsymbol{D}$ where $\boldsymbol{A} \in R^{r \times s}$, $\boldsymbol{B} \in R^{r \times (n-s)}$, $\boldsymbol{C} \in R^{(m-r) \times s}$, $\boldsymbol{A} \in R^{(m-r) \times (n-s)}$. Then the matrices $\boldsymbol{B}$, $\boldsymbol{C}$ and $\boldsymbol{D}$ could be used to predict $\boldsymbol{A}$. Specifically if both $\boldsymbol{X}$ and $\boldsymbol{D}$ have rank k then $\boldsymbol{A} = \boldsymbol{B}\boldsymbol{D}^{+}\boldsymbol{C} = \boldsymbol{B}(\widehat{\boldsymbol{D}}^{k})^{+}\boldsymbol{C}$ [14], where $\boldsymbol{D}^{+}$ represents the pseudoinverse of $\boldsymbol{D}$ and $\widehat{\boldsymbol{D}}^{k}$ represents the k-rank approximation of D obtained by Singular Value Decomposition. Using this property we partitioned the rows and columns of $\boldsymbol{X}$ respectively into h and l subsets so that each $h \times l$ subset represented a different hold out matrix $\boldsymbol{A}$. Finally we estimated the Bi-Cross Validation error as function of the k-rank approximation of the $\boldsymbol{D}$ matrices as:

$$BCV(k) = \sum_{i=1}^{h} \sum_{j=1}^{l} A_{i,j} - B_{i,j} \left(\widehat{D}_{i,j}^{(k)} \right)^{+} C_{i,j}{}_{F}^{2} \qquad \text{(Equation 5)}$$

By systematically changing k we expect the error would reach its minimum around the true rank of $\boldsymbol{X}$.

Stimulus-response specificity

The Specificity Index (SI) for each behavioral response was estimated as the weighted fraction nearest neighbor responses evoked by the same stimulus class. A formal definition of this index is given as follows. Let each ith behavioral response be quantified by its projection X_i on the R^d space of the first d principal components. We define the distance between each pair of responses as $dist_{ij} = X_i - X_{j}{}_{L2}$ and its inverse $w_{ij} = 1/dist_{ij}$. The K-neighborhood of each target response is then defined as the K responses associated with the smallest pairwise distances. Let each ith response be also associated with a variable $Y_i = \{1, 2\}$ representing the stimulus class. In this way each ith response is defined by the pair $(X_i, Y_i) \in R^d \times \{1, 2\}$. We can then define SI_i, the Specificity Index for the ith response as:

$$SI_i = \frac{\sum_{j=1}^{K} w_{ij} I(Y_i = Y_j)}{\sum_{j=1}^{K} w_{ij}} \qquad \text{(Equation 6)}$$

Where the indicator function $I()$ is equal to 1 if $Y_i = Y_j$ and 0 otherwise.

Decoding Analysis

Decoding performances for K-Nearest Neighbor (KNN) and Random Forest were estimated by using 10-fold cross-validation. Dimensionality reduction based on Principal Component Analysis was performed on the data before training the classifiers. To maximize performances the KNN algorithm was run by systematically varying the parameter K and the number of Principal Components (Figures S6A and S6B) while the Random Forest algorithm was run by systematically varying the number of Trees (within the set [10, 20, 40, 80, 160, 320]) and the number of Principal Components. Each tree was constrained to express a maximum number of 20 branches. For robustness, the estimates of decoding performances for both KNN and Random Forest were repeated 50 times for each parameter combination. Data and source codes for specificity and decoding analyses can be found here:
https://github.com/RStorchi/HighDimDefenseBehaviours/tree/master/Decoding

Clustering and Information Analysis

Clustering was performed by using k-means algorithm with k-means++ initialization [57]. The number of clusters k was systematically increased in the range (2-30). For each value of k, clustering was repeated 50 times and for each repeat the best clustering results was selected among 100 independent runs. We then used Shannon's Mutual Information to estimate the statistical dependence between response clusters and stimuli. A similar approach has been previously applied to neuronal responses (see e.g., [58–60]). In order to estimate Shannon's Mutual Information the probabilities distributions $p(G)$ and $p(G|S)$, where $\mathbb{G} = (g_1, \ldots, g_k)$ indicates the cluster set and $\mathbb{S} = (s_1, \ldots, s_n)$ the stimulus set, were estimated directly from the frequency histograms obtained from our dataset. Thus for

$p(G)$ we counted the number of elements in each cluster and we divided by the overall number of elements. We estimated $p(G,S)$ in the same way and used it to estimate $p(G|S)$ as $p(G,S)/p(S)$. From these distributions the response and noise entropies were calculated as

$$H(G) = -\sum_{g \in G} p(g) log_2 p(g) \quad \text{(Equation 7)}$$

$$H(G|S) = -\sum_{g \in G,\, s \in S} p(g,s) log_2 p(g|s) \quad \text{(Equation 8)}$$

These naive estimates were then corrected for the sampling bias by using quadratic extrapolation as in [61]. Mutual Information (*MI*) was then calculated from the difference of these corrected estimates. The change in *MI* as function of the number of clusters was fit by using *Equation 2* through a mean square error minimization based on the interior point method (MATLAB function *fmincon*). For fitting the values of the parameters a, b and τ were constrained to be positive. Data and source code for clustering analysis can be found here:

https://github.com/RStorchi/HighDimDefenseBehaviours/tree/master/Diversity

Analysis of Behavioral Primitives

Behavioral primitives were first identified by applying kmeans++ clustering ([57], best of n = 100 replicates for each parameter combination) to the response matrix. For this analysis the response matrix encompassed an epoch starting 0.33 s before the stimulus onset and ending 2 s after the onset. Since both the number of clusters and the duration of the primitive was unknown we repeated the clustering for a range of [2, 10] clusters and for six different durations (0.133 s, 0.2 s, 0.333 s, 0.4 s, 0.666 s and 1 s). In order to model arbitrarily (finite) long temporal relations between subsequent primitives occurring on the same trial we used Variable-order Markov Models (VMMs [62, 63],). Therefore an additional parameter of this analysis was represented by the maximum Markov order that ranged from 0 (no statistical dependence between two subsequent primitives), to the whole length L of the trial (L = 15, 10, 6, 5, 3 and 2 for primitives of 0.133 s, 0.2 s, 0.333 s, 0.4 s, 0.666 s and 1 s duration). To determine the best VMMs we took a decoding approach. This enabled us to rank the models according to their accuracy in predicting the stimulus on hold-out data. For each combination of cluster cardinality, primitive duration and maximum Markov order we trained three VMMs, one for each stimulus (flash, loom and sound). Thus each of the three VMM (respectively $VMM_{\mathbf{flash}}$, $VMM_{\mathbf{loom}}$, $VMM_{\mathbf{sound}}$) was separately trained by using a lossless compression algorithm based on Prediction by Partial Matching [64] on a subset of trials associated with only one stimulus. On the test set the stimulus $\widehat{S}$ was then decoded by choosing the VMM with highest likelihood $\widehat{S} = argmax_{stim\ \varepsilon\ \{flash,\ loom,\ sound\}}(VMM_{stim})$. Increasing the temporal resolution of the model by using a larger number of shorter duration primitives increased decoding accuracy (Figure S7A). Parameters for the eight most accurate models are reported in Figure S7B. Data and source code for VMMs analysis can be found here:

https://github.com/RStorchi/HighDimDefenseBehaviours/tree/master/VMMs

Clustering Refinement

To test the possibility that the "one-to-many" mapping shown in Figure 7B arise from incorrect cluster membership we developed a procedure to improve goodness-of-clustering. The element of each cluster were ranked according to their distance from the centroid. Then for each centroid we removed up to 50% of its elements according to such distance. This resulted in improved clustering metrics as shown in Figures S7D and S7E.

Estimating the Effects of Initial Positions

Initial positions were quantified according to 5 dimensions: head elevation and azimuth, and head X,Y,Z coordinates. In order to partition the space of initial conditions we first generated a set of 5 elements arrays with up to 8 partitions (each partition with the same number of trials) for each dimension (e.g., [1, 3, 4, 1, 1] indicates 3 and 4 partitions respectively along the 2nd and 3rd dimension) . For each array in this set the overall number of partitions across the 5 dimensions was the product of the number of partitions in each dimension (e.g., equal to 12 for the previous example). From the initial set we then removed all the items with an overall number of partitions larger than 20. Mutual Information was then estimated for each partition array as described in Clustering and Information Analysis. Finally a linear extrapolation was performed to estimate Mutual Information in the limit of an infinite number of partitions.

Current Biology, Volume *30*

Supplemental Information

A High-Dimensional Quantification of Mouse Defensive Behaviors Reveals Enhanced Diversity and Stimulus Specificity

Riccardo Storchi, Nina Milosavljevic, Annette E. Allen, Antonio G. Zippo, Aayushi Agnihotri, Timothy F. Cootes, and Robert J. Lucas

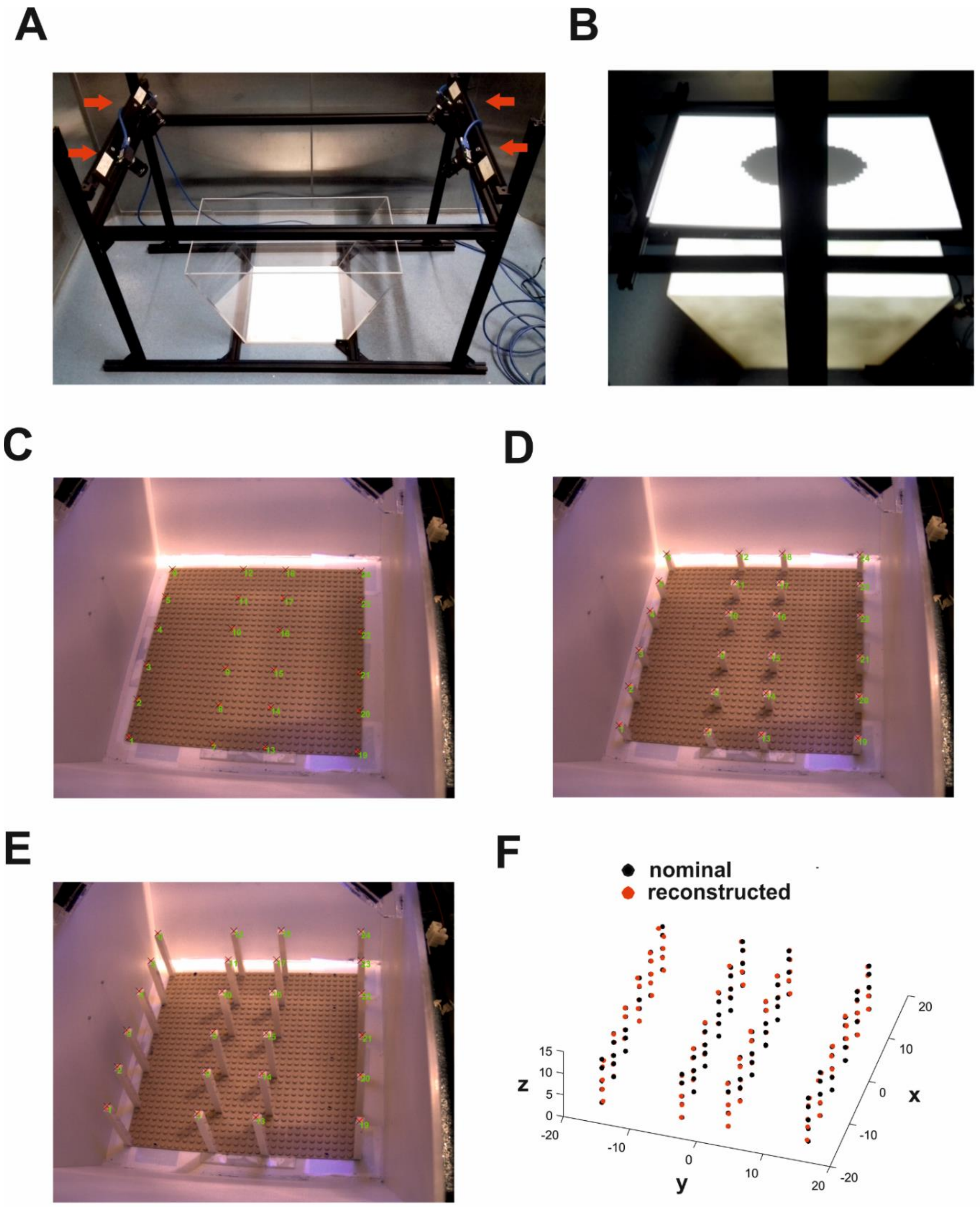

Figure S1: Calibration of 4 camera system for 3D reconstruction related to Figure 1A and STAR Methods. A) A picture of our system. The behavioural arena is placed in the centre and cameras are indicated by red arrows. **B)** Visual stimuli, such as the standard black looming disc, are presented through a rear-projection screen. **C)** To calibrate the cameras we used 24 landmarks on a Lego® plate that tiled a large portion of the arena. These landmarks were repeated at 5 different heights by using Lego® bricks as shown in panels **D** and **E. F)** After calibration the 3D reconstructed position of the landmarks (red dots) matched the nominal positions (black dots).

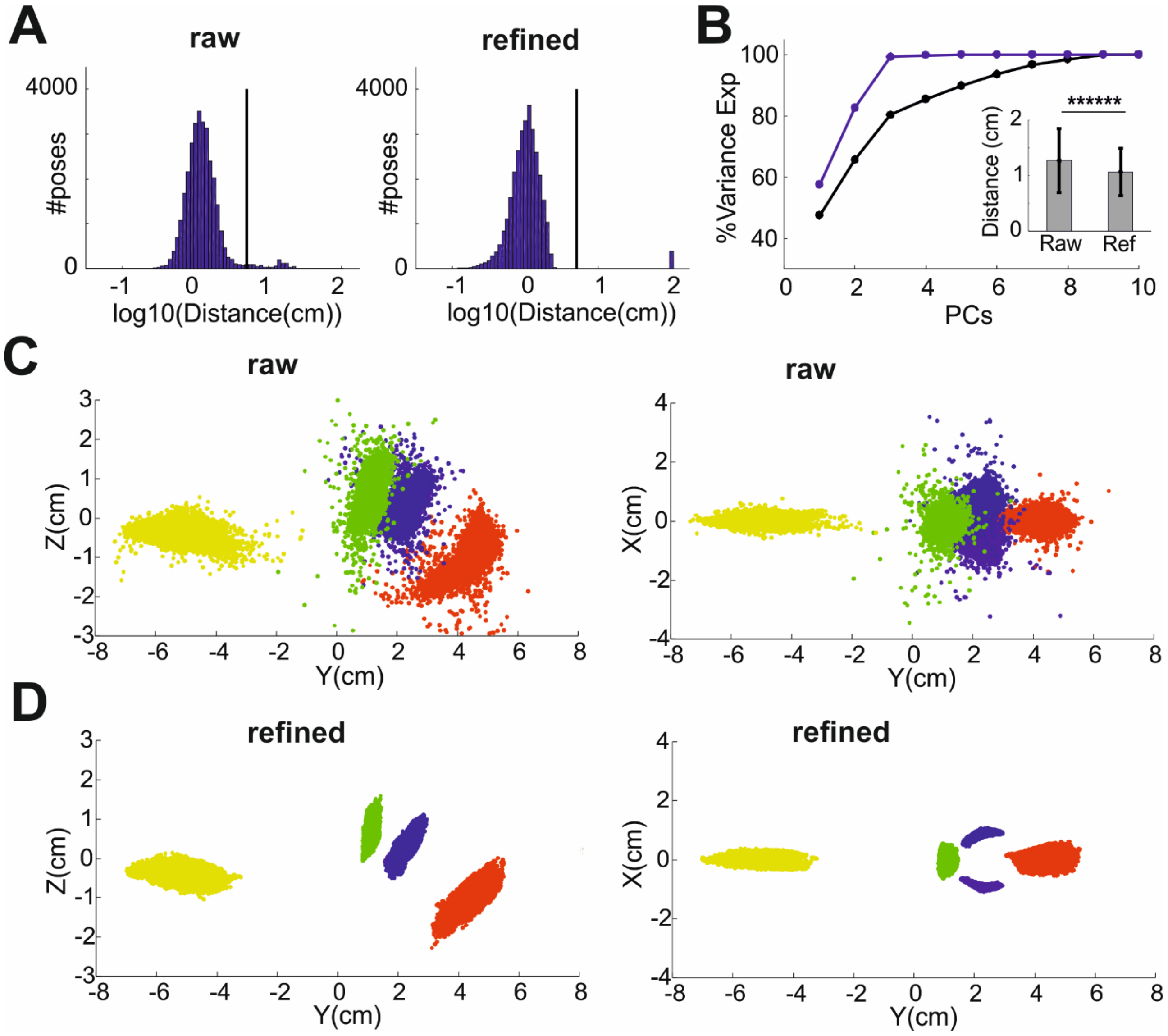

Figure S2: Validation of the 3D reconstruction related to Figure 1B and STAR Methods. A) Distribution of Euclidean distances between all poses and the mean pose for raw and refined 3D reconstruction (respectively left and right panel). The threshold that separates outlier and inlier poses is indicated by black vertical lines. B**)** Percentage of variance explained by a Principal Component Analysis of raw and refined 3D poses (respectively blue and black lines). **C)** Full dataset of aligned poses obtained via the raw 3D reconstruction. The 3D poses are shown from a side and top view (respectively left and right panel). **D)** Same as in panel **C** but for the refined 3D reconstruction.

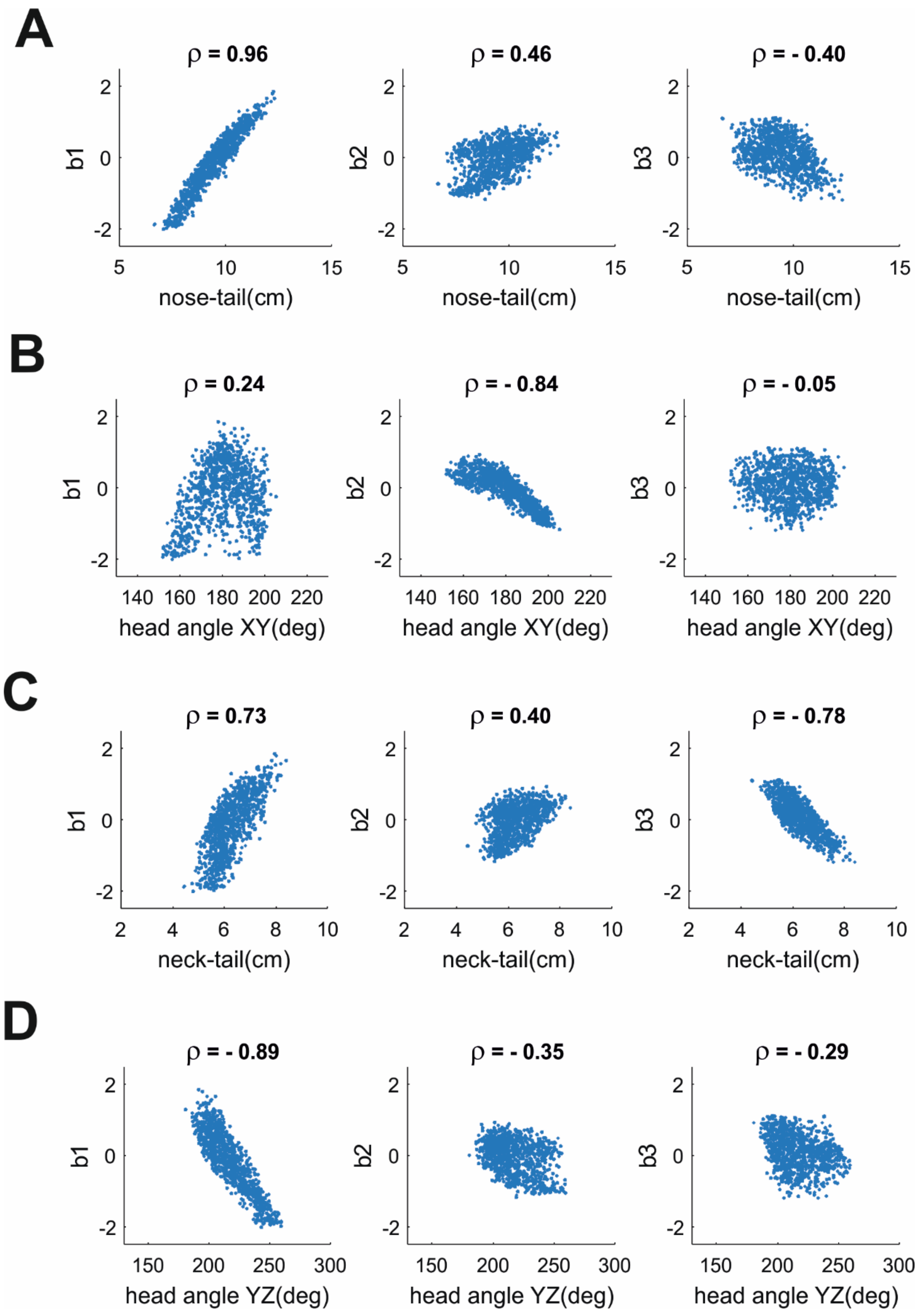

Figure S3: interpretation of the eigenposes related to Figure 1C&D. A) Shape parameters b1, b2 and b3 are plotted as function of nose-tail distance. **B-D)** Same as panel a but here shape parameters are plotted as function of head-angle on the XY plane (**B**), neck-tail distance (**C**) and head-angle on the YZ plane (**D**). Pearson's correlation values are reported at the top of each panel.

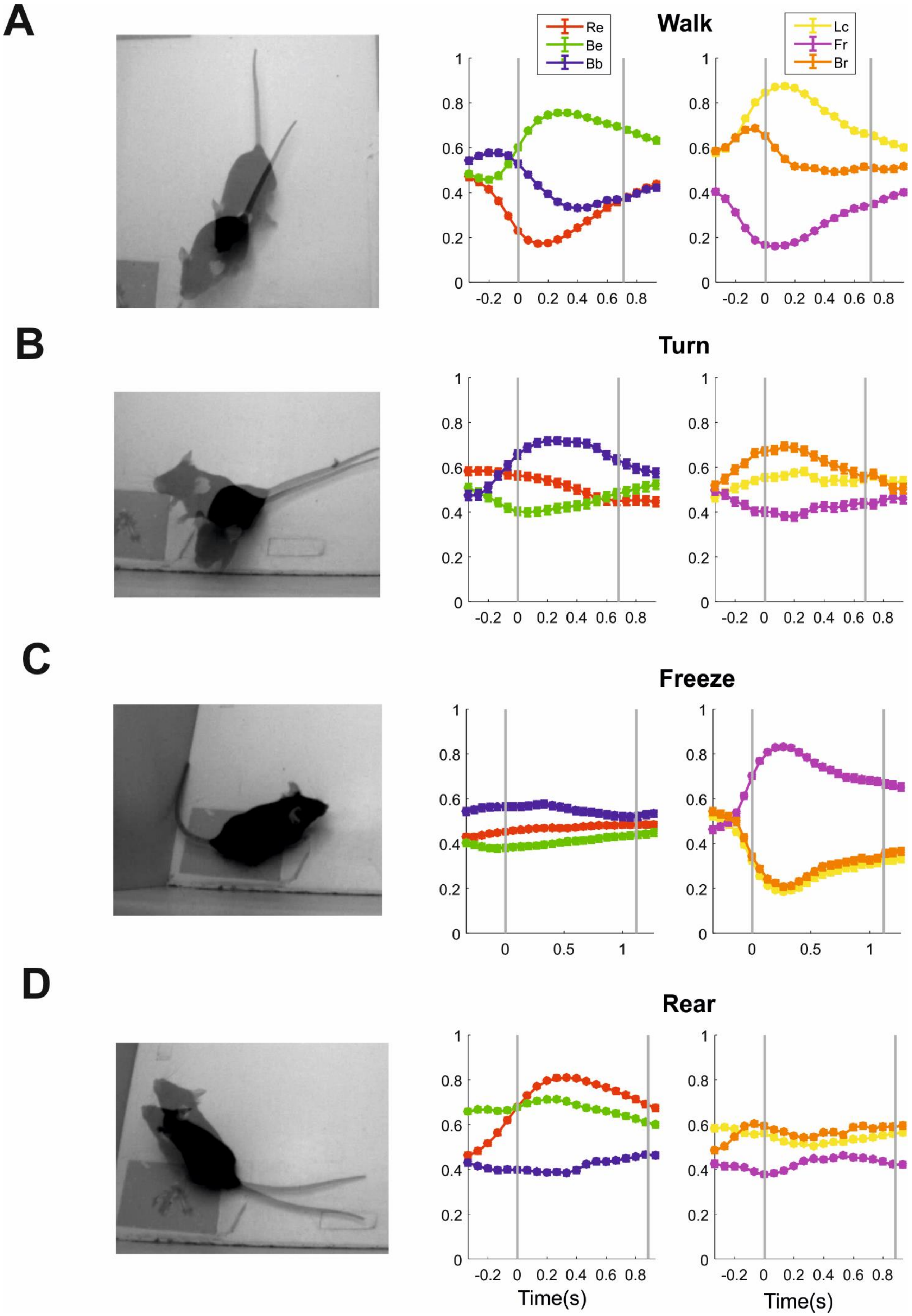

A
Walk
Re
Be
Bb
Lc
Fr
Br
B
Turn
C
Freeze
D
Rear
Time(s)
Time(s)

Figure S4: Validation of the postural and movement measures related to Figure 1D and STAR Methods. Manually annotated actions “Walk”, “Turn”, “Freeze” and “Rear” are compared with postural measures of Rearing, Body Elongation and Body Bending (Re, Be and Bb in centre panels) and movement measures of Locomotion, Freezing and Body Rotation (Lc, Fr and Br in left panels). Right panels show representative samples of two superimposed frames 0.267 seconds apart. Centre and left panels show mean±sem of each measure (n = 473, 214, 371 and 505 for Walking, Turning, Freezing and Rearing). In each panel the two vertical grey lines indicate the beginning (at time 0) and average duration of each action.

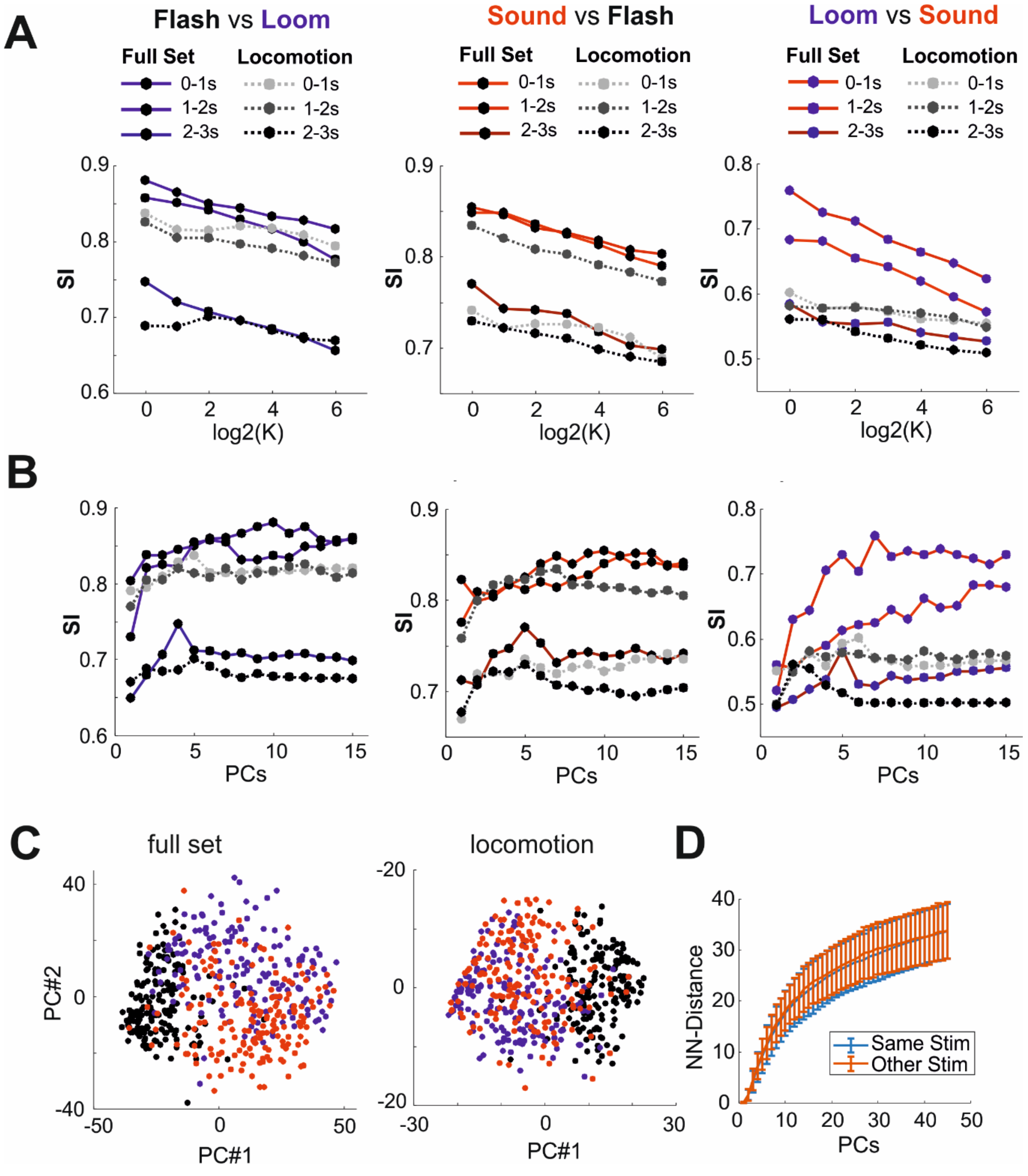

Figure S5: Higher dimensionality reveals increased specificity in sensory guided behaviours related to Figure 3B&C. A) Specificity Index is shown as function of the number of Principal Components for the full set (continuous lines) and for locomotion only (dashed lines). Different response epochs are represented in different levels of brightness (bright: 0-1s; intermediate: 1-2s; dark: 2-3s). **B)** Same as panel **A** but here SI is shown as function of K. **C)** Scatterplot of the first two Principal Components for full set and locomotion only (respectively left and right panel). Each dot correspond to an individual trial and is coloured according the stimulus (black, blue and red for Flash, Loom and Sound respectively). **D)** Euclidean distance (Mean±SD) of the nearest neighbours to target trials. Blue and Red error bars indicate respectively those trials in which the nearest neighbours was associated with the same (blue) or with a different stimulus (red). None of the 45 pairwise comparisons between the two groups is significant at 0.05 after applying rank-sum tests with Bonferroni correction.

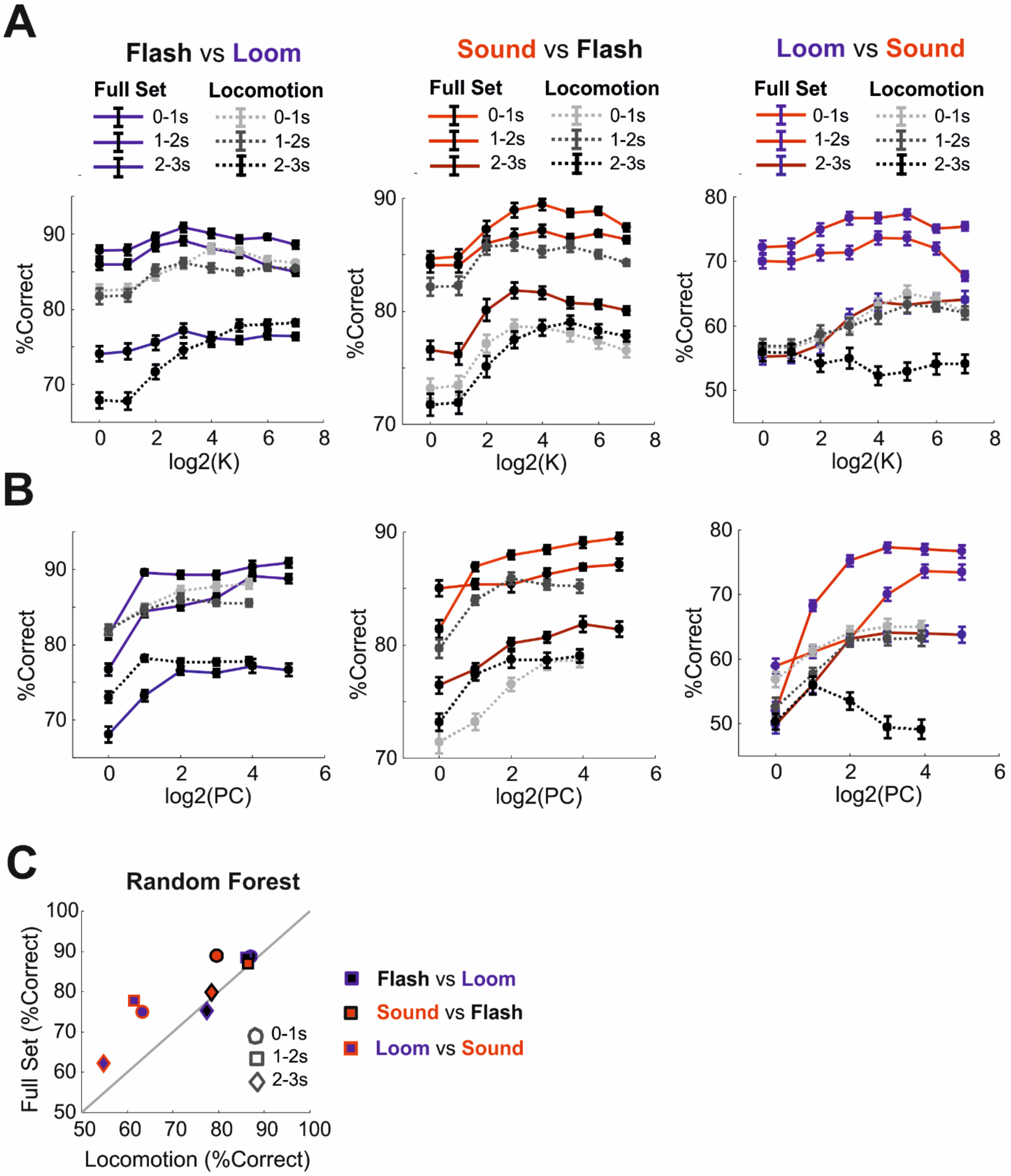

Figure S6: Higher dimensionality improves stimulus decoding related to Figure 4A. A) Stimulus decoding performances across a range of K values for the full set (continuous lines) and for locomotion only (dashed lines). Different response epochs are represented in different levels of brightness (bright: 0-1s; intermediate: 1-2s; dark: 2-3s). **B)** Same as panel a but here the performances are shown as function of the number of Principal Components used for decoding. **C)** Comparison between Random Forest decoding performances based on the full set and on locomotion only. Pairwise comparisons are shown for flash vs loom (black-blue), sound vs flash (red-black) and loom vs sound (blue-red) across different response epochs (0-1s, 1-2s, 2-3s).

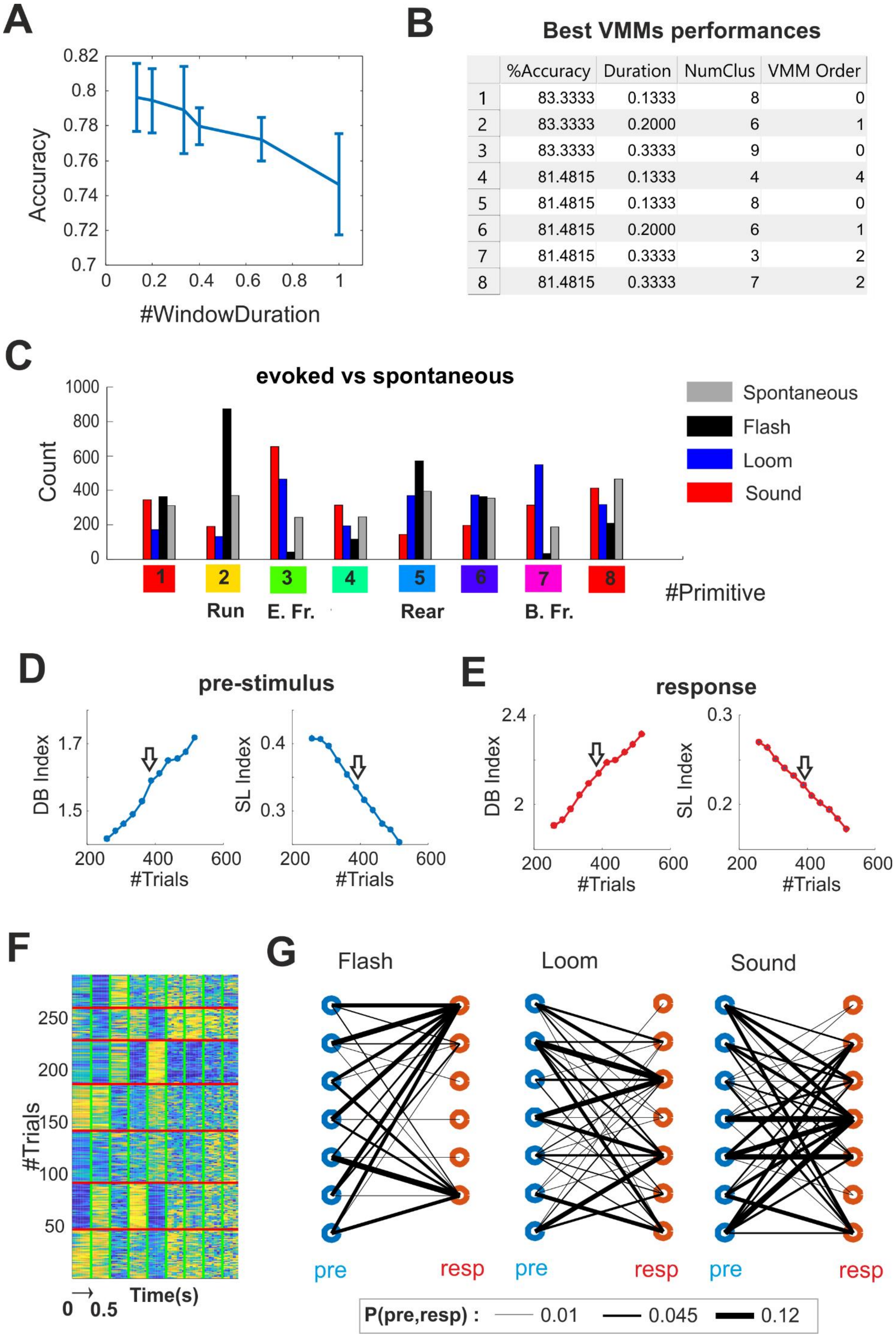

	%Accuracy	Duration	NumClus	VMM Order
1	83.3333	0.1333	8	0
2	83.3333	0.2000	6	1
3	83.3333	0.3333	9	0
4	81.4815	0.1333	4	4
5	81.4815	0.1333	8	0
6	81.4815	0.2000	6	1
7	81.4815	0.3333	3	2
8	81.4815	0.3333	7	2

Figure S7: Distinct behaviours differ both in rate and latency of behavioural primitives related to Figure 6A&B and Figure 7A&B. A) Decoding performances of the VMMs as function of primitive duration (Mean±SD for the 10 best models of each duration). **B)** Defining parameters (Primitive Duration, Number or Clusters, maximum order of VMMs) for the 8 best VOMMs. **C)** Distribution of the 8 primitives for spontaneous activity preceding the stimulus and across responses to flash, loom and sound. **D)** Davies-Bouldin (DB) and Silhouette (SL) indexes as function of the number of pre-stimulus trials. Subset of trials are gradually removed from the full dataset in order to increase goodness-of-clustering as measured by decreasing DB and increasing SL indexes. **E)** Same as panel **D** for response trials. **F,G)** Same as **Fig.7A&B** but here we consider only those trials associated with better DB and SL indexes for pre-stimulus and response (indicated in panels **D** and **E** by arrows).
